# Hydrotalcite Colloidal Stability and Interactions with Uranium(VI) at Neutral to Alkaline pH

**DOI:** 10.1021/acs.langmuir.1c03179

**Published:** 2022-02-15

**Authors:** Chris Foster, Samuel Shaw, Thomas S. Neill, Nick Bryan, Nick Sherriff, Louise S. Natrajan, Hannah Wilson, Laura Lopez-Odriozola, Bruce Rigby, Sarah J. Haigh, Yi-Chao Zou, Robert Harrison, Katherine Morris

**Affiliations:** † Research Centre for Radwaste Disposal and Williamson Research Centre, Department of Earth & Environmental Sciences, The University of Manchester, Oxford Road, Manchester M13 9PL, U.K.; ‡ National Nuclear Laboratory, Chadwick House, Warrington Road, Birchwood Park, Warrington WA3 6AE, U.K.; § Centre for Radiochemistry Research, Department of Chemistry, The University of Manchester, Oxford Road, Manchester M13 9PL, U.K.; ∥ Sellafield Ltd., Hinton House, Birchwood Park Avenue, Risley, Warrington, Cheshire WA3 6GR, U.K.; ⊥ Department of Materials, The University of Manchester, Oxford Road, Manchester M13 9PL, U.K.; # Nuclear Fuel Centre of Excellence, Department of Mechanical, Aerospace and Civil Engineering, The University of Manchester, Sackville Street, Manchester M13 9PL, U.K.

## Abstract

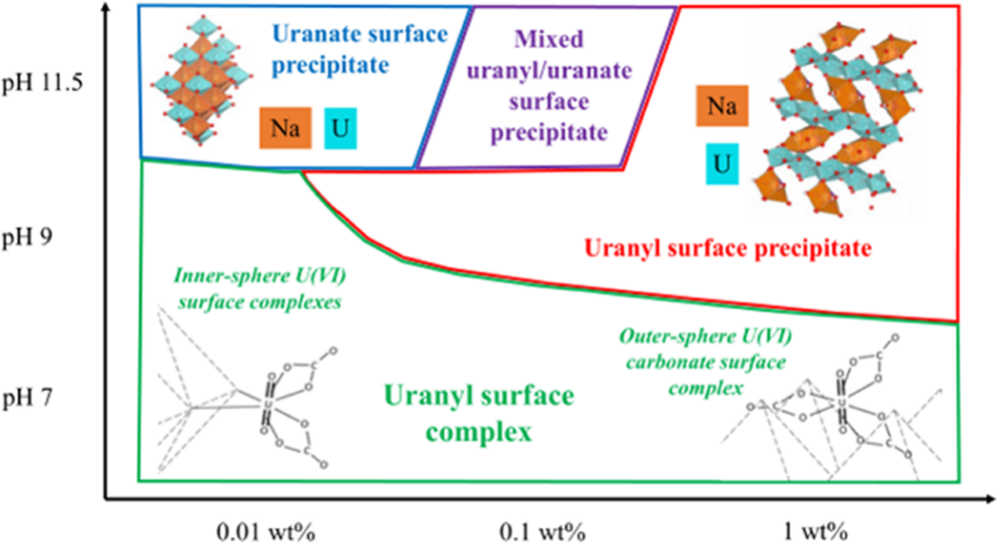

In the United Kingdom, decommissioning of legacy spent fuel storage facilities involves the retrieval of radioactive sludges that have formed as a result of corrosion of Magnox nuclear fuel. Retrieval of sludges may re-suspend a colloidal fraction of the sludge, thereby potentially enhancing the mobility of radionuclides including uranium. The colloidal properties of the layered double hydroxide (LDH) phase hydrotalcite, a key product of Magnox fuel corrosion, and its interactions with U(VI) are of interest. This is because colloidal hydrotalcite is a potential transport vector for U(VI) under the neutral-to-alkaline conditions characteristic of the legacy storage facilities and other nuclear decommissioning scenarios. Here, a multi-technique approach was used to investigate the colloidal stability of hydrotalcite and the U(VI) sorption mechanism(s) across pH 7–11.5 and with variable U(VI) surface loadings (0.01–1 wt %). Overall, hydrotalcite was found to form stable colloidal suspensions between pH 7 and 11.5, with some evidence for Mg^2+^ leaching from hydrotalcite colloids at pH ≤ 9. For systems with U present, >98% of U(VI) was removed from the solution in the presence of hydrotalcite, regardless of pH and U loading, although the sorption mode was affected by both pH and U concentrations. Under alkaline conditions, U(VI) surface precipitates formed on the colloidal hydrotalcite nanoparticle surface. Under more circumneutral conditions, Mg^2+^ leaching from hydrotalcite and more facile exchange of interlayer carbonate with the surrounding solution led to the formation of uranyl carbonate species (e.g., Mg(UO_2_(CO_3_)_3_)^2–^_(aq)_). Both X-ray absorption spectroscopy (XAS) and luminescence analysis confirmed that these negatively charged species sorbed as both outer- and inner-sphere tertiary complexes on the hydrotalcite surface. These results demonstrate that hydrotalcite can form pseudo-colloids with U(VI) under a wide range of pH conditions and have clear implications for understanding the uranium behavior in environments where hydrotalcite and other LDHs may be present.

## Introduction

Uranium (U) is typically the most abundant radionuclide by mass in both the nuclear fuel cycle and many radioactive waste inventories.^1,2^ It is also a contaminant associated with uranium mining and where accidental release of radionuclides to the environment has occurred.^3,4^ Understanding the speciation and mobility of uranium in interim storage, waste processing, and contaminated land is important in underpinning safe decommissioning and management of operations at nuclear facilities. The mobility and lability of uranium in aqueous systems is controlled by its chemical speciation. Under oxic conditions, U(VI) is present at ambient pH as the uranyl UO_2_^2+^_(aq)_ ion, which at neutral to elevated pH forms soluble carbonate species (e.g., UO_2_(CO_3_)_2_^2–^_(aq)_).^5^ Under more alkaline conditions or higher U concentrations, the solubility of U(VI) can be limited due to precipitation of uranyl phases (e.g., compreignacite (Na_2_(UO_2_)_6_O_4_(OH)_6_·7H_2_O))^6,7^ and/or uranate compounds (Na_2_UO_4_, Na_2_U_2_O_7_).^8−10^ The mobility and lability of U(VI)_(aq)_ can also be controlled by its sorption to solid phases. While extensive work has been performed on U(VI) sorption to solids, characterization of U(VI) interactions with colloids, which may enhance the mobility of U(VI), has received less focus. Crucially, understanding the sorption of U(VI) to hydrotalcite (Mg_6_Al_2_CO_3_(OH)_16_·4H_2_O) colloids is key to understanding the behavior of this radionuclide in a range of systems, for example, fuel pond environments and effluent treatment processes.

With decommissioning of global facilities including Sellafield’s legacy fuel storage facilities and the retrieval of waste scheduled to take place over the next few decades, the behavior of U(VI) and hydrotalcite colloids under spent fuel ponds and effluent treatment conditions is of high interest as several studies have confirmed that colloids exist in legacy fuel ponds and storage facilities.^11−14^ The legacy storage facilities at Sellafield include spent nuclear fuel ponds (SNFPs), which range between circumneutral and alkaline pH (7–11.5) and have highly heterogeneous inventories, which include SNF, cementitious materials, microbes, pond furniture, and extraneous debris.^15−17^ A potential source of colloidal phases within these legacy fuel storage facilities is waste sludge, which has built up during extended storage and subsequent corrosion of spent nuclear fuel and cladding.^18,19^ Within SNFP at Sellafield, UK, the waste sludge is typically composed of uranium metal fuel elements and magnesium-based cladding sourced from UK Magnox reactors, which have corroded over time.^20^ Previous studies have identified brucite (Mg(OH)_2_) and hydrotalcite as key phases that result from cladding corrosion.^21−23^ Studies have shown that brucite can form radionuclide-doped pseudo-colloids (e.g., ^90^Sr, Am, and Pu).^14,24^ Hydrotalcite, a layered double hydroxide (LDH), has a structure composed of brucite-like layers and interlayer regions containing interchangeable anionic species. While recent studies have investigated the aggregation behavior, nanoparticle structure, and intercalation properties of colloidal hydrotalcite,^25−28^ there is a paucity of information regarding its colloidal stability across the pH range 7–11.5. This is despite the fact that hydrotalcite has been identified as a key phase in many nuclear treatment systems (e.g., nuclear fuel storage facilities) and radioactive wastes/contaminated environments as a solid phase and/or colloidal particles.^22,29−31^ Furthermore, it has been established that LDHs and nanoparticulate metal (oxyhydr)oxides are effective at sequestering U(VI) in a range of scenarios via a variety of adsorption, incorporation, and intercalation processes and therefore are likely to be key controls on the mobility and fate of U(VI) in these environments.^32−38^ A study investigating the surface charge properties of colloidal hydrotalcite indicated that the nanoparticles carried positive charges under circumneutral to alkaline pH (6–11), with the charge magnitude seen to decrease as the pH increased, indicating that colloidal stability was reduced under more alkaline conditions.^39^ Furthermore, previous studies have examined the affinity and adsorption mechanisms of U(VI) with Mg–Al-based LDHs.^40−42^ These studies showed that hydrotalcite possessed a high adsorption capacity (up to >99% uptake) for uranium within the studied pH range of 3–9.5. However, the molecular scale mechanisms of U(VI) uptake (e.g., adsorption vs surface precipitation) to LDHs under conditions relevant to nuclear fuel storage and effluent treatment environments, and the impacts of variable U(VI) concentrations and pH on those interactions, are poorly constrained.^17^ Previous X-ray absorption spectroscopy (XAS) and X-ray photoelectron spectroscopy studies suggest that at near-neutral and weakly basic pH, U(VI) forms inner-sphere surface complexes, although the mechanism by which these surface complexes form and the potential impacts of both pH and variable U(VI) concentration are poorly constrained.^38,43,44^ Song et al. showed that the sorption of U(VI) to LDHs involved ligand–exchange reactions between surface hydroxyl groups and UO_2_^2+^ at pH 5, while at pH 8, carbonate anions, which were removed from the hydrotalcite interlayers due to exchange with other anionic species, had an increased role in the formation of U(VI) surface complexes.^44^ Gräfe et al., while studying the sequestration of U(VI) by hydrotalcite under circumneutral and alkaline conditions, found that under CO_2_-rich conditions, uranyl sorbed to the hydrotalcite as an inner-sphere U(VI)–carbonate complex.^38^ By contrast, other workers have identified outer-sphere complexation of U(VI) with Mg-based minerals, for example, brucite.^45^ Further research is required to clarify the mechanism(s) of U(VI) sorption to hydrotalcite under neutral to basic conditions with changing U(VI) concentration. Understanding these interactions will aid in underpinning effluent treatment technologies within nuclear fuel pond storage facilities as well as in other systems where hydrotalcites may be present, for example, the treatment of uranium mining effluents, radioactive waste disposal in cementitious environments, and the remediation of contaminated land.^30,46^ Additionally, understanding the sorption behavior of U(VI) onto hydrotalcite has the potential to inform on the behavior of transuranics, notably plutonium, a key radiological component of many waste streams.^13,47,48^

Here, a multi-technique approach was adopted to investigate the stability of colloidal hydrotalcite in the presence and absence of U(VI) using ultrafiltration and zeta potential analysis. In addition, the U(VI) sorption mechanisms between pH 7 and 11.5 as a function of U(VI) concentration/surface loading were characterized using a combination of XAS, luminescence spectroscopy, and geochemical modeling. Results showed that hydrotalcite remained colloidal across the studied pH range, with U(VI) sorbing readily under all conditions. Spectroscopic data showed that the U(VI) adsorption mechanism was highly dependent on pH and U(VI) concentration/surface loading.

## Experimental Section

### Colloidal Hydrotalcite Preparation

Colloidal hydrotalcite was synthesized following the method of Xu et al.^49^ Briefly, two solutions, one containing both magnesium and aluminum chloride hexahydrate (0.30 and 0.10 M, respectively) and the other containing sodium hydroxide (0.15 M) and sodium carbonate (0.015 M), were mixed in a 1:1 v/v ratio at room temperature and stirred for 10 min to yield a hydrotalcite slurry with a Mg/Al ratio of 3:1. The resultant slurry was then collected via centrifugation, washed twice, and dispersed in deionized water before being hydrothermally treated at 100 °C for 16 h.^49^ These hydrothermal conditions were chosen so as to yield hydrotalcite nanoparticles with a narrow particle size distribution (∼100 nm in diameter), as has been outlined by Xu et al.^49^ BET analysis (Micromeritics Gemini VII) of the hydrotalcite yielded a surface area of 76.1 ± 2.1 m^2^/g. Following this, the pH of the resultant colloidal suspension (pH 9.3–9.5) was adjusted in batch experiments to pH 7, 8, 9, 10, 11, and 11.5 (within 0.1 pH unit) using either 2.5 M NaOH or 5 M HNO_3_. Finally, NaCl was added to yield a final ionic strength of 10 mM in all experiments. The colloidal systems were kept in sealed bottles over the course of a month and were uncapped during sampling. At selected time points, Mg and Al concentrations in the colloidal suspension were measured by dissolving aliquots of the suspension in 2% HNO_3_, followed by inductively coupled plasma atomic emission spectroscopy (ICP–AES) analysis to check the concentration of Mg and Al and thus the stability and reproducibility of the colloidal synthesis between batches. The average concentration of the hydrotalcite, all of which was present in the batch experiments as a suspended colloidal phase at the start of the experiments, was 5 g/L, with the standard deviation for the Mg and Al concentrations across different batches calculated as ±6.3%.

### U(VI) Adsorption Experiments

To investigate the sorption mechanisms of U(VI) to hydrotalcite, hydrotalcite colloids were prepared at different pHs (7–11.5) and spiked with varying U(VI) concentrations of 2.1, 21, and 210 μM. These concentrations corresponded to U(VI) loadings (assuming essentially complete sorption) of 0.01, 0.1, and 1 wt % on the colloidal hydrotalcite at 5 g/L. Uranium as U(VI) was spiked from a pH 1 U(VI) nitric acid stock, and the pH was then adjusted to 7, 8, 9, 10, 11, and 11.5 (±0.1) using either NaOH (2.5 M) or HNO_3_ (5 M). NaCl was then added to yield a final ionic strength of 10 mM in all experiments. Again, the U(VI)-hydrotalcite colloidal experiments were kept in sealed bottles over the course of a month and were uncapped during sampling.

### Particle Size and Colloidal Stability Characterization

The effects of pH and U(VI) loading on the rate of colloid settling were tracked using Imhoff settling cones.^50^ Here, for colloidal stability experiments, 100 mL of hydrotalcite colloidal suspension (5 g/L) at pHs 7, 8, 9, 10, 11, and 11.5 was transferred to the cones, which were then sealed, and the settleable solid volume was recorded over 1 month. Similarly, for U(VI) loading experiments, 100 mL of 5 g/L colloidal hydrotalcite suspension at pH 11.5 with the three different U(VI) loadings (2.1, 21, and 210 μM) was transferred to the sealed cones, and the settleable solid volume was recorded over 1 month.

To assess colloidal particle size distributions, ultrafiltration analysis was carried out on 200 mL suspensions of colloidal hydrotalcite (5 g/L) both with and without U(VI) addition. The size distributions were analyzed in 0.5 mL samples routinely taken from 2 cm below the air–water meniscus after 1 h, 1 week, 2 weeks, and 1 month. The samples (0.5 mL) were filtered using Pall Nanosep poly(ether sulfone) centrifugation (ultra)filters with pore sizes of 3 kDa (approximately 1.5 nm), 0.2 and 0.45 μm.^51−53^ Filtrations were conducted via filter centrifugation at 7000*g* for 20 min to determine the concentrations of Mg, Al, and U in the “dissolved” (<1.5 nm), “smaller colloidal” (1.5 nm to 0.2 μm), “larger colloidal” (0.2–0.45 μm), and “coarse” (>0.45 μm) filtered fractions. The total U concentration in the filtered, acidified (2% HNO_3_) samples was measured by ICP–MS (Agilent 7500cx), while the total Mg and Al concentrations were measured using ICP–AES (PerkinElmer Optima 5300 dual view).

The effect of pH on hydrotalcite surface charge was explored using zeta potential measurements. Colloidal hydrotalcite systems with various pHs (pH 7, 8, 9, 10, 11, and 11.5) were synthesized at the FENAC facility (University of Birmingham). One milliliter aliquots were sampled at 2 cm below the meniscus for the experiments after 1 h, 1 week, 2 weeks, and 1 month. The samples were then analyzed using a Malvern ZetaSizer at 25 °C with a He–Ne laser (λ = 633 nm) measuring backscattered light at 173° and disposable TS1060 zeta potential and sizing cuvettes, with three scans taken for each sample.

Selected colloidal samples were then prepared for transmission electron microscopy (TEM) analysis. The samples were removed from the pH 9 0 wt % U(VI) and the pH 7 1 wt % U(VI) colloidal suspensions (at 24 h and 2 cm below the meniscus), pipetted onto carbon-coated gold TEM grids (Agar Scientific), and gently washed with ethanol (2 × 0.5 mL) and deionized water (2 × 0.5 mL) prior to analysis.^54^ To obtain the colloidal particle size distributions, bright-field TEM images were taken from the samples using a FEI TF30 TEM. To understand the U(VI) adsorption sites, high-angle annular dark-field (HAADF) STEM images were taken using a FEI Titan G2 S/TEM.

### Structural and U(VI) Adsorption Mechanistic Characterization

To identify any changes to the nanoparticle structure from changing either pH or the U(VI) loading, X-ray diffraction (XRD) analysis was conducted on hydrotalcite samples collected from colloidal suspensions at varying pHs (7, 8, 9, 10, 11, and 11.5) and U(VI) loading (2.1, 21, and 210 μM) at 1 h, 1 week, 2 weeks, and 1 month. The colloidal hydrotalcite particles were collected using centrifugation (9600*g*, 20 min) and dried in a vacuum desiccator for 24 h. Powder XRD data was recorded using a Bruker D8Advance with Cu Kα X-rays (wavelength 1.5406 Å), with Si used as a standard for all samples. Hydrotalcite colloidal particles for experiments without U(VI) were collected using centrifugation and analyzed using a PerkinElmer Spotlight 400 FTIR imaging system with an ATR accessory across the wavenumber range of 500–4000 cm^–1^ in order to explore the impact of pH change on nanoparticle structure.

For X-ray absorption spectroscopy (XAS) analysis, hydrotalcite colloids were prepared containing U(VI) loadings of either 2.1, 21, and 210 μM U(VI) at pHs 7, 9, and 11.5. After 24 h equilibration at constant pH, the suspended material was collected as a wet solid paste for analysis via centrifugation (9600*g*, 20 min). The sample was then mounted in a cryogenic cell and frozen at −80 °C prior to analysis and transport to Diamond Light Source. Data collection was carried out using a liquid nitrogen cryostat at beamlines B18 (36-element Ge detector) or I20 (64-element Ge detector) at the U L_III_-edge in the transmission mode (pH 7, 9, and 11.5, [U] = 210 μM) or the fluorescence mode (pH 7, 9, and 11.5, [U] = 2.1 and 21 μM).^55^ Energy calibration was performed using the first inflection point of a yttrium foil (17,038 eV), with between 10 and 25 scans collected and averaged for each sample. The data were analyzed using the Demeter software packages Athena and Artemis, FEFF6, with the data typically fit to k and r space ranges of 3–12 and 1–4.5, respectively.^56^

For luminescence analysis, hydrotalcite colloids were prepared containing U(VI) loadings of either 2.1 μM (0.01 wt %) or 210 μM (1 wt %) on the hydrotalcite colloidal suspensions (assuming complete sorption to the colloid) at pH 7, 9, and 11.5. After 24 h equilibration at constant pH, the colloidal suspension (0.75 mL) was sampled at a 2 cm depth and immediately flash-frozen as a suspension using liquid N_2_. The steady-state emission spectra were recorded on an Edinburgh Instruments FP920 phosphorescence lifetime spectrometer equipped with a liquid N_2_ finger Dewar, a 450 W steady-state xenon lamp (with a single 300 mm focal length excitation and emission monochromators in Czerny Turner configuration), and a red sensitive photomultiplier in a Peltier (air cooled) housing (Hamamatsu R928P) detector.^9,57^ Each scan was run in triplicate at −198 °C using an excitation wavelength of 280 nm. For pH 11.5 and pH 7, 0.01 wt % samples, the steady-state spectra were weak and dominated by background fluorescence, so a 0.1 ms time delay was applied. Lifetime data were recorded following excitation with a 5 W μs xenon flash lamp and a multi-channel scaling method, with gate times applied during emission measurements.^57^ Lifetimes were obtained by tail fits of the data, with the quality of the fits judged by optimizing the reduced χ^2^ and minimizing the residuals squared; the decay profiles fitted best to double-exponential decays in all cases. The percentages quoted with the lifetime values in the following sections refer only to the % of the total emission intensity and are not representative of the relative proportions of different sorption modes, as the quantum yields of emission will be different for different sorption modes.

Thermodynamic modeling of the experiments was performed using PHREEQC version 3.3.5 using a modified ThermoChimie Database.^58^ Here, the equilibrium constant for the aqueous Mg(UO_2_(CO_3_)_3_)^2–^_(aq)_ species was added to the database from the literature.^59^
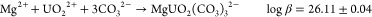


For each input file, the Mg and Al concentrations, which were informed from the ICP–AES analysis of the batch experiments, were equilibrated with hydrotalcite. This was done to model any predicted Mg and Al leaching, which may in turn affect the U(VI) aqueous speciation.

## Results and Discussion

### Hydrotalcite Nanoparticle Structure and Colloidal Stability

XRD analysis was conducted on solid samples collected from hydrotalcite colloidal systems of varying pH and U(VI) loading (Figure S1). All samples yielded XRD patterns with characteristic peaks indicative of an ordered, crystalline hydrotalcite structure and with no evidence for crystalline U-bearing phases (Table S1).^60,61^ For the pH 7 and 8 samples, additional peaks in the XRD patterns indicated the presence of a small quantity of gibbsite (Al(OH)_3_) (Figure S2).^62^ Over the course of a month, apart from in the 1 wt % U(VI) samples, there were no significant changes in the diffraction patterns. In the 1 wt % U(VI) samples at all pH, the hydrotalcite (003) peak position showed small but systematic shifts to higher *d*-spacing, suggesting that a small expansion of the interlayer distance of between 0.03 and 0.05 Å occurred (Figure S3). Song et al., who observed similar shifts in the interlayer distance when investigating U(VI) sorption onto ternary LDHs, suggested that this was a result of interlayer carbonate having a higher binding affinity toward U(VI) than the surface hydroxyl groups of hydrotalcite. This enables the formation of U(VI)–carbonate complexes and enhances the exchange of interlayer carbonate for other anionic species such as OH^–^ and NO_3_^–^, resulting in slight increases in the interlayer spacing.^44^ In addition, there was also noticeable broadening of the (003) peak for the pH 7, 8, and 9, 1 wt % samples (Figure S4). This indicates that there is greater variation in the average interlayer distance, which is likely due to the increased disorder that results from the OH–CO_3_^2–^ ligand exchange between the hydrotalcite hydroxyl surface groups and the interlayer carbonate.^44^ This exchange process is enhanced under circumneutral conditions as the interlayer carbonate will be HCO_3_^–^, which has a reduced electrostatic affinity for the positively charged hydrotalcite interlayer surfaces compared to CO_3_^2–^ and so will be more readily exchanged for other anionic species present in the system such as OH^–^ and NO_3_^–^.^63−65^

Fourier transform infrared (FTIR) analysis was undertaken to identify the key anions within the hydrotalcite interlayer, as well as to observe any potential changes in the structure with pH and time. Overall, the data across all the experiments were consistent, showing characteristic peaks which match those previously identified for carbonate-intercalated hydrotalcite.^66^ These peaks include the C–O stretch of the interlayer carbonate anions at ∼1360 cm^–1^, the O–H stretch of the interlayer H_2_O molecules at ∼1638 cm^–1^, and the O–H stretches and bending of the surface hydroxyl groups at ∼3360 cm^–1^ (Table S2 and Figure S5). When comparing the carbonate peaks across the spectra, in the pH 7 and 8 systems, the position was shifted to lower wavelengths (1353 cm^–1^) and a shoulder feature at 1400 cm^–1^ was present (Figure S6). These changes are due to a loss in the *D*_3*h*_ symmetry of the carbonate anions within the interlayers as the carbonate changes from CO_3_^2–^ to HCO_3_^–^ under circumneutral conditions.^63−65,67^

**Figure 1:**
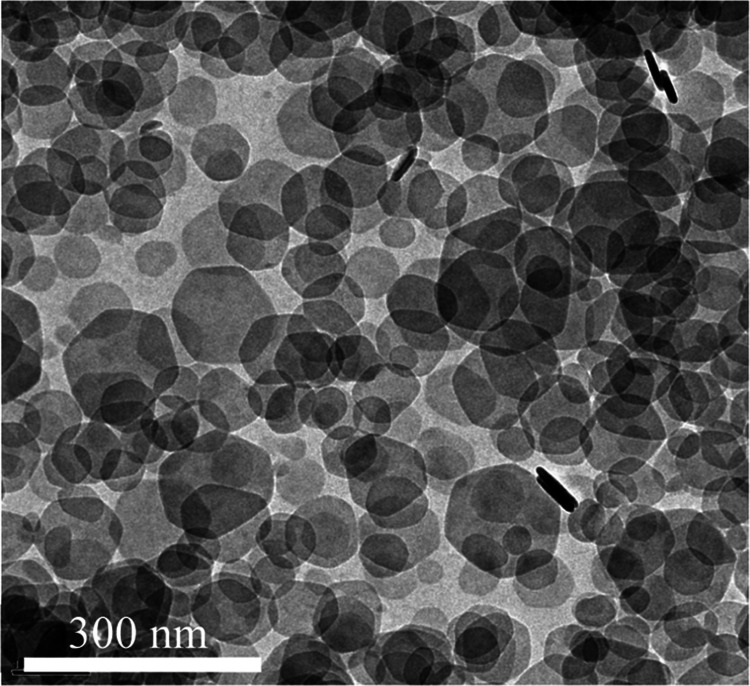
Bright-field TEM images of hydrotalcite nanoparticles taken from the pH 9 colloidal suspension after 1 week.

TEM imaging of samples taken from the pH 9 experiment after 1 week showed well-dispersed hydrotalcite platelets with a hexagonal morphology, an average diameter of approximately 90 nm (Figure 1), and a thickness of approximately 10 nm.

**Figure 2:**
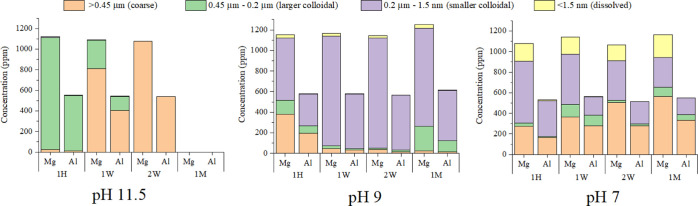
Ultrafiltration data for the experiments without U(VI) present and highlighting the changes in hydrotalcite colloidal particle size distribution in the supernatant with varying pH over the course of a month: 1 h (1 H), 1 week (1 W), 2 weeks (2 W), and 1 month (1 M). The different size ranges are the coarse (>0.45 μm), larger colloidal (0.2–0.45 μm), smaller colloidal (1.5 nm to 0.2 μm), and dissolved (<1.5 nm) fractions.

To explore the impact of pH on the settling rates and particle size distribution of colloidal hydrotalcite, settling cone and ultrafiltration experiments were performed without U(VI) present. No decrease in the 100 mL settleable solid volume of the hydrotalcite colloid was observed after 1 month at pH between 7 and 11 (Figure S7). At pH 11.5, the settleable solid volume showed a steady decrease from 100 mL at 3 days to 21 mL after 1 month (Figure S7). Ultrafiltration-ICP-AES data for Mg and Al concentrations in hydrotalcite colloidal suspensions are highlighted in Figure 2, with the complete dataset provided in Figures S8–S13. In the pH 11.5 experiments, there was an increase in the proportion of Mg and Al in the coarse size fraction (>0.45 μm) over 2 weeks (3% at 1 h to 100% at 2 weeks) (Figure S8). After a month, the colloid–liquid interface had descended below the point of sampling, as observed with the settling cone data. By contrast, for the pH 9 and pH 10 systems, the majority (>54 and >56%, respectively) of the Mg and Al and thus colloids were measured within the smaller colloidal size fraction (0.2 μm to 1.5 nm) at all time points (Figures 2, S10, and S11). The proportion of Mg in the dissolved fraction (<1.5 nm) increased from 3% at pH 9 to 14–17% at pH 7, suggesting that leaching of Mg^2+^ from the hydrotalcite occurred. At pH 8, Mg and Al were measured in all size fractions over the course of a month with no clear trends in the data with time (Figure S12). By comparison, the pH 7 system showed a continued increase in the proportion of Mg and Al in the coarse size fraction (>0.45 μm) with time (from 31% at day 1 to 51% at 1 month), suggesting aggregation of particles (Figure S13).

Zeta potential measurements were also taken at 1 h, 1 week, 2 week, and 1 month time points for the different pH systems to track the changes and variation in colloidal stability. The average zeta potential magnitudes for each of the different pH experiments are shown in Figure S14. The average zeta potential magnitude for the pH 11.5 system was the lowest for all pH at +35 ± 3.0 mV. There were then systematic increases in the average zeta potential magnitude for the pH 11 (+43 ± 3 mV), pH 10 (+50 ± 3 mV), and pH 9 systems (+54 ± 2 mV) as the pH decreased (Figure S14). However, for the pH 8 and pH 7 experiments, there was no increase in the average zeta potential magnitude when compared to the pH 9–11.5 systems, with values of +54 ± 5 and +49 ± 4 mV for the pH 8 and 7 systems, respectively, despite the pH being shifted further from the pH_PZC_ of hydrotalcite (pH_PZC_ ∼ 12.2) (Figure S14).

Collectively, results from the settling, ultrafiltration, and zeta potential analyses have shown that between pH 7 and 11.5, the synthesized hydrotalcite colloids remained suspended for several weeks. For the pH 11.5 system where the zeta potential values were lowest (Figure S14), the ultrafiltration data showed a continued increase in the proportion of particles in the coarse size fraction (>0.45 μm) during the first 2 weeks, while the settling experiment showed a substantial decrease in the settleable solid volume over 2 weeks. This is a result of the hydrotalcite nanoparticles aggregating more readily at pH 11.5 as they carry a lower net positive charge compared to the lower pH systems. At pH 10 and 9, the shift away from the pH_PZC_ means the hydrotalcite nanoparticles carry larger positive charges and therefore experience greater interparticle repulsion, reducing their propensity for aggregation. This behavior is evidenced in the ultrafiltration data as the majority of the particles were measured in the small colloidal size fraction (0.2 μm to 1.5 nm). For the pH 8 and 7 systems, the ultrafiltration results showed an increasing proportion of particles in the higher size fractions, suggesting aggregation due to lower colloidal stability. This occurred despite the pH of these experiments being further from the pH_PZC_ of hydrotalcite of 12.2. One explanation for the decrease in colloidal stability observed was leaching of Mg^2+^ from the hydrotalcite structure, as evidenced by the systematic increase in dissolved Mg^2+^ at lower pH (Figures 2 and S11–S13). Here, leaching of Mg^2+^ from the nanoparticles altered the hydrotalcite surface structure and led to the formation of gibbsite as observed in the XRD data for pH 8 and 7 experiments (Figure S2).^68^ This in turn will impact the colloidal properties of the nanoparticles with the pH_PZC_ trending to a value more typical of gibbsite (pH_PZC_ ∼ 8.35).^69^ Given the pH 8 and 7 experiments are closer to this gibbsite-like pH_PZC_, the positive charge on the surface of the colloidal particles will be reduced, which enables flocculation. This is indicated by the zeta potential analysis with the pH 8 (+54 ± 5 mV) and 7 (+49 ± 4 mV) systems not having larger zeta potential magnitudes than the pH 9 colloidal system, despite the pH being further from the hydrotalcite pH_PZC_ (Figure S14).

### U(VI) Sorption to Colloidal Hydrotalcite

Ultrafiltration and settling experiments were conducted over the course of 1 month in the presence of U(VI) and at variable pH (7–11.5) to determine the effect of U(VI) loading on the settling rates and particle size distribution of colloidal hydrotalcite.

**Figure 3:**
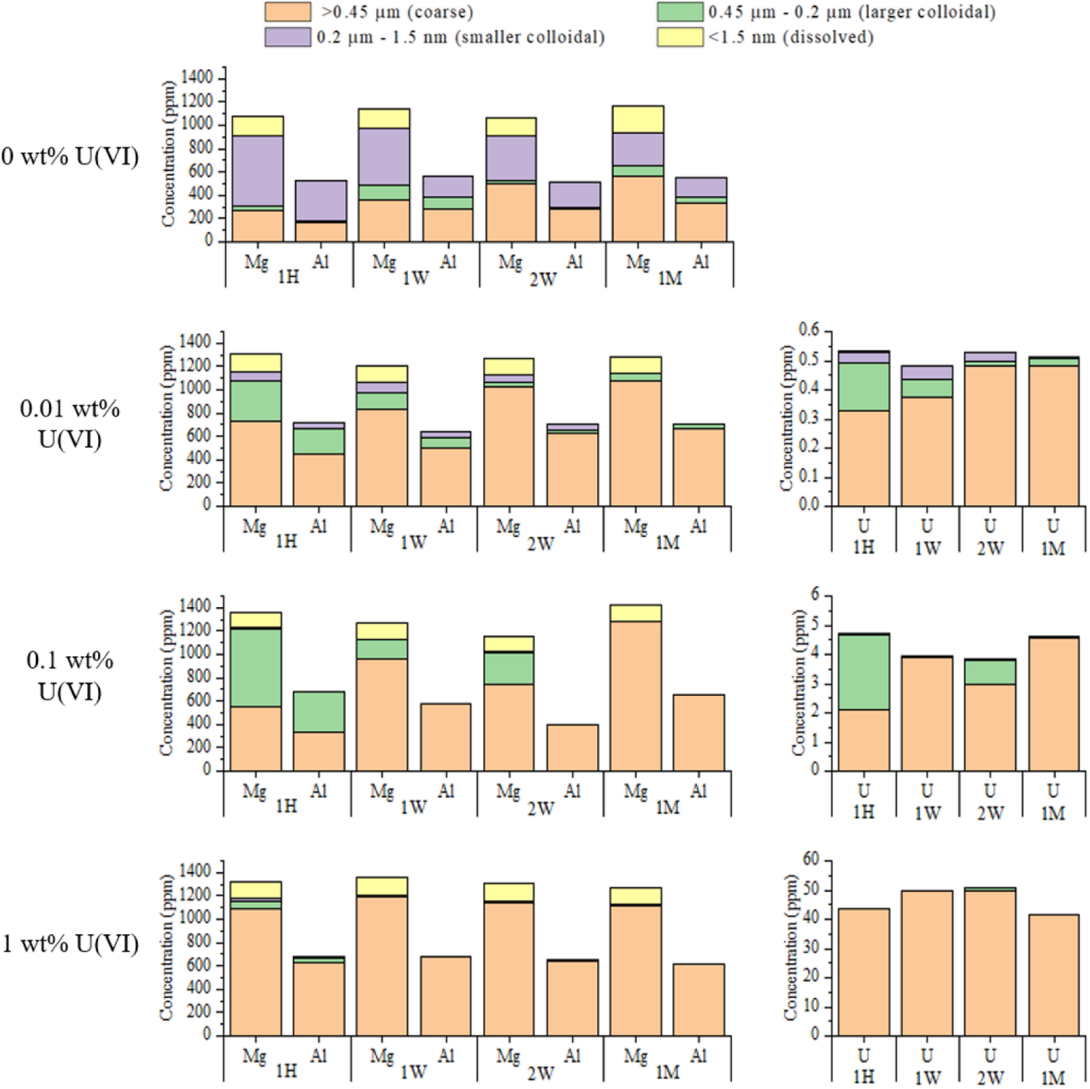
Ultrafiltration data showing Mg, Al, and U concentrations in the different ultrafiltration fractions with time and highlighting the changes in the hydrotalcite colloidal particle size distributions with variable U(VI) loading for the pH 7 system over the course of a month.

The ultrafiltration results indicated that varying the pH between 7 and 11.5 had no significant effect on the extent of U(VI) sorption, with greater than 98% of U(VI) sorption onto colloidal particles in all systems, irrespective of pH, U(VI) loading, or time (Figures S8–S13). The distribution of uranium in each size range in the ultrafiltration data showed close correlation with the distribution of both Mg and Al, confirming that U(VI) sorption occurred uniformly to hydrotalcite across the colloidal systems (Figures S8–S13). Overall, the data suggested that with increased U(VI) loading, aggregation of the colloidal hydrotalcite particles to larger sizes was promoted (for the pH 7 system, see Figure 3 and for pH 8–11.5, see Figures S8–S13). At pH 7, ≤56% of Mg and Al was detected in the larger colloidal (0.45–0.2 μm) and coarse (>0.45 μm) size fractions over the course of a month in the absence of any U(VI) (Figure 3). However, as the U(VI) loading was increased, the proportion of Mg and Al measured in the larger colloidal (0.45–0.2 μm) and coarse (>0.45 μm) size fractions increased to >80, >87, and >89% at U(VI) loadings of 0.01, 0.1, and 1 wt %, respectively, confirming U facilitated aggregation. Indeed, similar trends were observed for the remaining pH experiments (pH 8–11), with increased U loading from 0.01 to 1 wt % resulting in increased proportions of Mg and Al concentrations in the larger colloidal (0.45–0.2 μm) and coarse (>0.45 μm) fractions (Figures S8–S13). In addition, Figures S15 and S16 show the effects of variable U(VI) loadings on the settling rate at pH 11.5 over 1 month. At all three U(VI) loadings, there was no change in the initial settleable solid volume over 24 h, but in the 1 wt % U(VI) system, the volume decreased significantly to 58 mL over 2 weeks. The 0.1 wt % and the 0.01 wt % U(VI) systems showed smaller reductions in their settling rates with volumes of 70 and 73 mL, respectively, at 2 weeks. The increased settling observed with increased U(VI) concentration is presumably due to the higher U(VI) loadings, reducing the positive charge on the surface of the colloid, either through the formation of surface precipitates or surface complexes, which in turn promotes flocculation (Figures S15 and S16).

### U(VI) Sorption Mechanism(s)

To determine the mechanism by which U(VI) sorbed to the hydrotalcite nanoparticles at pH 7, 9, and 11.5, a combination of analytical techniques were used. Collectively, XAS data, luminescence analysis, PHREEQC modeling, and TEM imaging suggested that the U(VI) sorption mechanism(s) were subject to pH and concentration effects, with the adsorption mechanisms outlined as follows:

- Uranyl oxyhydroxide surface precipitates formed at pH 9 (U(VI) loadings of 1 and 0.1 wt %) and pH 11.5 (U(VI) loadings of 1 wt %)
- Uranate-like surface precipitates formed at pH 11.5 as the U(VI) surface loading was reduced to 0.01 and 0.1 wt %
- Uranyl surface complexes were the dominant sorption mode for the pH 9, 0.01 wt % U(VI) system and for all pH 7 U(VI) surface loading systems

These three sorption mechanism are discussed in detail in the following sections.

### X-ray Absorption Spectroscopy

**Figure 4:**
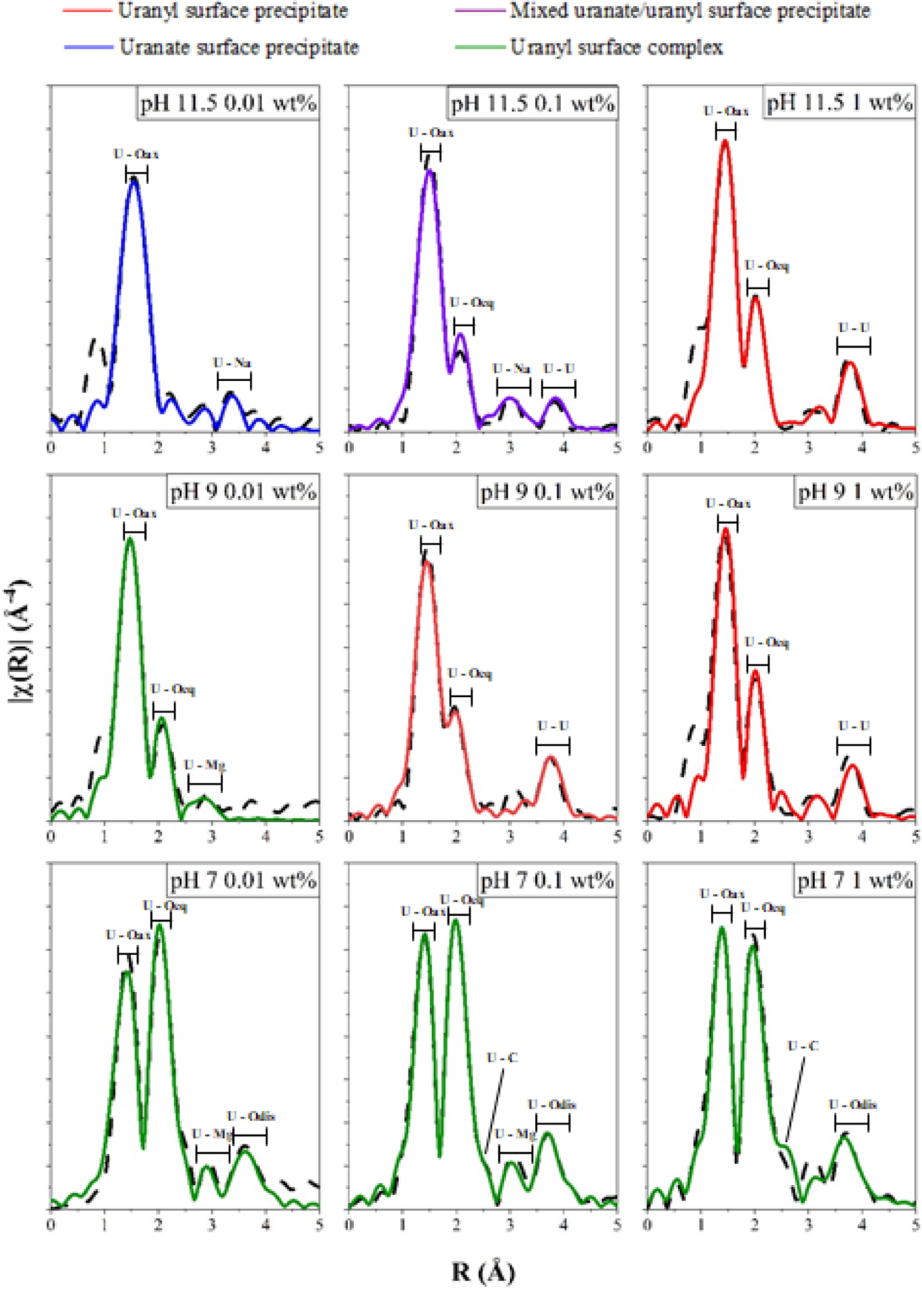
Fourier transform of k^3^-weighted EXAFS for the nine studied XAS samples. The black dashed lines show experimental data. The colored lines show the fits described in Tables S3–S5. Labels are included to highlight features attributed to the axial U–O uranyl shells (U–O_ax_), uranium–oxygen equatorial shells (U–O_eq_), uranium–carbon shells (U–C), uranium–magnesium shells (U–Mg), uranium–sodium shells (U–Na), and uranium–uranium shells (U–U).

Uranium XAS analyses were conducted on samples from hydrotalcite colloids reacted with U(VI) at pH 7–11.5 and loadings of 0.01–1 wt % 24 h after U(VI) addition. The Fourier transforms of the XAS data (black dashed lines) are shown in Figure 4 with the corresponding best fits (colored lines), with the complete dataset and fits provided in Figures S17–S20 and Tables S3–S5. A red fit line indicates the formation of uranyl surface precipitates, purple indicates the formation of a mixed uranyl–uranate surface precipitate, blue indicates the formation of a dominant uranate surface precipitate, and green indicates the dominance of uranyl surface complexes. Analysis of the XAS data were informed by published extended X-ray absorption fine structure (EXAFS) and crystallographic data for relevant U(VI) standards (Table S6).^10,70,71^

### Uranyl Oxyhydroxide Surface Precipitates

The EXAFS fits for the pH 11.5 1 wt % U(VI), pH 9 1 wt % U(VI), and pH 9 0.1 wt % U(VI) samples included two U–O axial backscatterers between 1.80 and 1.82 Å (Tables S3 and S4). For the pH 11.5 1 wt % U(VI) and pH 9 1 wt % U(VI) fits, the equatorial oxygen shell consisted of a total of six oxygen backscatterers, with 5 O between 2.21 and 2.44 Å and 1 O between 2.86 and 2.90 Å (Table S3). For the pH 9 0.1 wt % U(VI) fit, 5.5 equatorial oxygens which were modeled at 2.22 Å (*n* = 1.5), 2.36 Å (*n* = 2), and 2.47 Å (*n* = 2) (Table S4). For all the three fits, a U–U shell was included at 3.87 Å with a coordination number of 3 (Tables S3 and S4).

The best fits for the pH 11.5 1 wt % U(VI), pH 9 1 wt % U(VI), and pH 9 0.1 wt % U(VI) samples are similar to published EXAFS and XRD crystallographic data for the uranyl oxyhydroxide precipitate compreignacite (Table S6).^70,72^ The structure of uranyl oxyhydroxide precipitates consists of repeating U–O polyhedral units linked by equatorial oxygen ligands. In the case of crystalline compreignacite, these polyhedral units are pentagonal bipyramidal, which is consistent with the equatorial oxygen contributions in the modeled best fits for our samples.^72,73^ For the pH 9 0.1% U(VI) system, there was no reduction in the U–U shell coordination number and no increase in the Debye–Waller factor associated with the U–U shell when compared to the pH 11.5 and pH 9 1 wt % U(VI) best fits, confirming that a compreignacite-like phase had also formed despite the decrease in U(VI) loading. However, the XRD analysis shows no evidence of any peaks that can be attributed to compreignacite; thus, the surface precipitates that form likely have reduced long-range order.

**Figure 5:**
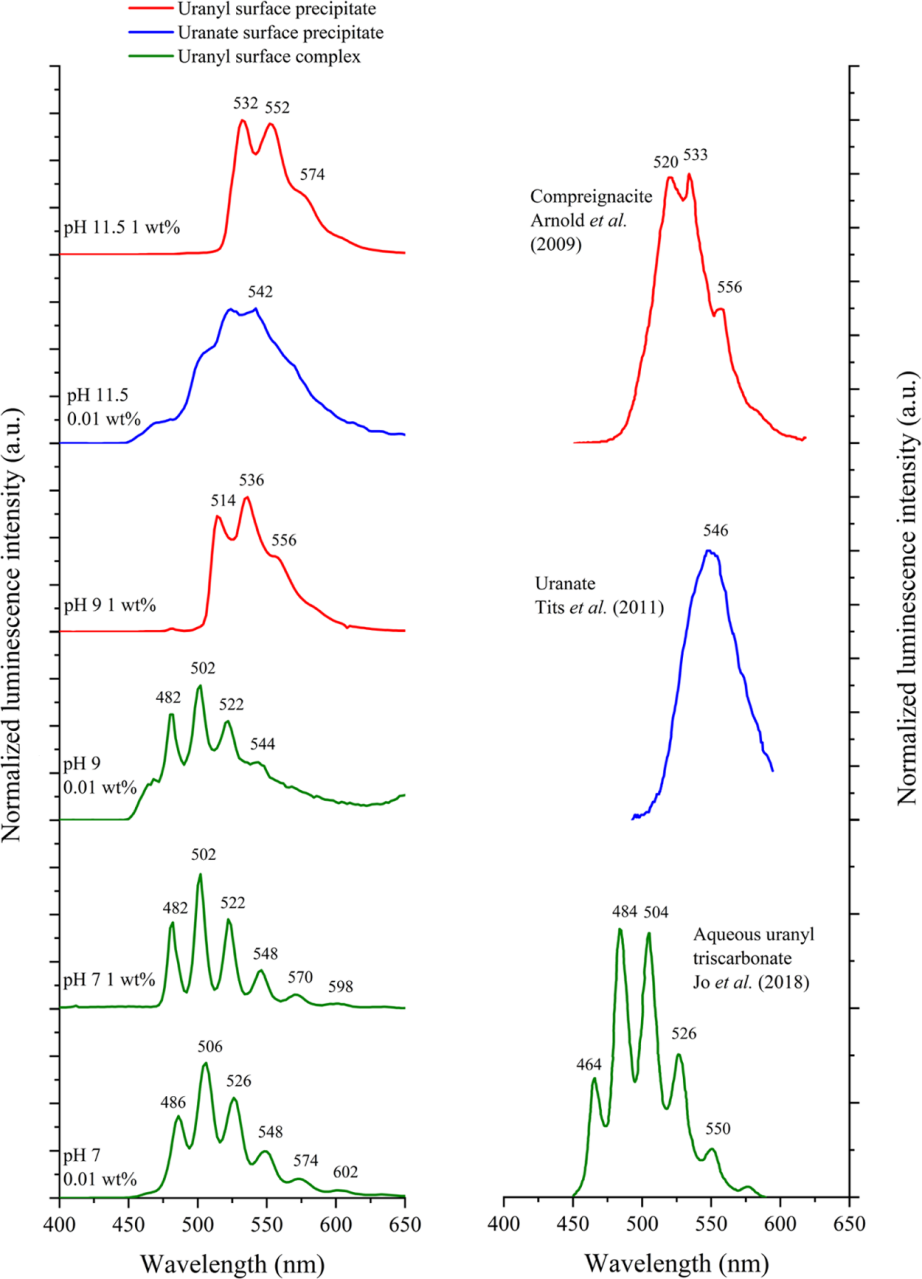
(Left) Normalized luminescence spectra for samples taken from hydrotalcite colloids with adjusted pHs of 11.5, 9, and 7 and with U(VI) loadings of either 1 wt % or 0.01 wt %. Spectra were recorded with an excitation wavelength of 280 nm and at 77 K on frozen solution samples. (Right) Published luminescence spectra for compreignacite, calcium uranate, and aqueous uranyl triscarbonate.

The luminescence spectra from the hydrotalcite colloids at pHs 11.5, 9, and 7 and U loadings of 0.01 and 1 wt % are provided in Figure 5 along with published luminescence spectra for compreignacite, calcium uranate, and aqueous uranyl triscarbonate.^74−76^ The key spectral features and luminescence lifetime values obtained via tail fitting are provided in Table S7. The luminescence spectrum for the pH 9 1 wt % U(VI) sample showed key peaks at 514 nm (19 455 cm^–1^) and 536 nm (18 657 cm^–1^), with a shoulder feature located at 556 nm (17 986 cm^–1^) (Figure 5).^77^ These positions are similar to those seen in previously published luminescence spectra for uranyl oxyhydroxide precipitates such as compreignacite (525, 543, and 565 nm), becquerelite (535, 556, and 581 nm), and schoepite (518, 540, and 565 nm).^74,75,78−81^ Fitting the lifetime data yielded two lifetime values of 137 ± 2 μs (47%) and 253 ± 2 μs (53%), which indicated the presence of two distinct coordination environments on the timescale of the experiment. This again suggests that the predominant U(VI) phase is compreignacite-like as it contains two symmetrically distinct U(VI) cations (and thus coordination environments), both of which are coordinated by two O atoms and three OH groups arranged at the equatorial corners of pentagonal bipyramids.^72^ Furthermore, the lifetime values are within the range of those reported for uranyl oxyhydroxide phases such as schoepite, meta-schoepite, becquerelite, and compreignacite (16–300 μs).^75,81^ These shorter lifetime values are a result of non-radiative processes, in particular, the dissipation of adsorbed energy throughout the extended lattice of the U(VI) precipitate.^82^

Thermodynamic PHREEQC modeling of the colloidal hydrotalcite experiments with different U(VI) loadings was undertaken to further interpret the data (Figures S21–S23). For the 1 wt % U(VI) system, sodium compreignacite (Na_2_(UO_2_)_6_O_4_(OH)_6_·7H_2_O) was the most oversaturated U(VI) phase at pH 9 (Figure S21) in agreement with both the EXAFS and luminescence data.

The luminescence spectrum for the pH 11.5 1 wt % U(VI) sample showed key peaks at 532 nm (18 797 cm^–1^) and 552 nm (18 116 cm^–1^) as well as a shoulder feature at 574 nm (17 422 cm^–1^), with distinct luminescence lifetime values of 69 ± 1 μs (24%) and 198 ± 1 μs (76%) (Figure 5).^77^ Indeed, the key spectral features for the pH 11.5 1 wt % sample were more red-shifted than those for the pH 9 1 wt % sample, with the latter closest to the published compreignacite spectra shown in Figure 5.^75,81^ This indicates that the pH 11.5 spectrum may be impacted by the presence of minor additional U(VI) phases; for example, both clarkeite and sodium uranate are oversaturated in the PHREEQC modeling results for the 1 wt % U(VI) system at pH 11.5 (Figure S21).

### Uranate Surface Precipitates

When the U(VI) loading was reduced at pH 11.5, the XAS data suggested a gradual transition from uranyl-like precipitates to uranate-like precipitates. As can be seen in Figure S18, the XANES spectra for the pH 11.5 1 wt % U(VI) sample shows a clear shoulder feature, which becomes less prominent and shifts to lower energies as the U(VI) loading is reduced to 0.1 and 0.01 wt %. This shoulder feature is diagnostic for uranyl speciation and becomes less prominent as the U(VI) loading falls and the local U–O coordination environment becomes more uranate-like.^70,83^

When considering the EXAFS data (Figure 4 and Table S5), the best fit for the pH 11.5 0.01 wt % U(VI) sample had 2 O backscatterers at 1.86 Å, a split equatorial O shell with 3 O backscatterers at 2.26 and 1.5 at 2.44 Å. The best fit also included 4 U–Na backscatterers at 3.31 and 3.54 Å but did not contain a U–U shell (Table S5).^9,75,78,84,85^ The elongated U–O axial bond length coupled with the inclusion of U–Na shells is consistent with the U(VI) coordination environment moving toward a uranate-like speciation.^8,10^ Sodium uranate is best described as having a perovskite-like structure, where elongated NaO_6_ and UO_6_ polyhedra alternate in position in the equatorial plane and are linked through oxygen bonds.^8^ The presence of two U–Na shells along with the absence of a U–U shell in the pH 11.5 0.01 wt % fit is consistent with this coordination environment.^8,86−88^ For the pH 11.5 0.1 wt % U(VI) sample, the fit was complex and included 2 O at 1.83 Å, 2.5 O at 2.26 Å, and 3 O at 2.45 Å as well as 1.3 U–Na backscatterers at 3.42 Å, which were statistically significant (using the *F*-test, Table S4).^89^ A U–U shell was also included at 3.92 Å. The shorter U–O axial bond length, the reduced overall U–Na shell coordination number, and the inclusion of a U–U shell when compared to the pH 11.5 0.01 wt % U(VI) fit suggested the presence of both uranyl oxyhydroxide and sodium uranate species.

The luminescence data also indicated a shift from uranyl-like to uranate-like precipitates as the U(VI) loading was reduced at pH 11.5 (Figure 5). When compared to the pH 11.5 1 wt % U(VI) sample, the luminescence spectrum for the 0.01 wt % sample was broader, relatively featureless, and had a lower relative emission intensity with the maximum intensity at 540 nm (18 519 cm^–1^) (Figure 5). The pH 11.5 0.01 wt % U(VI) spectrum is similar to published data for Ca–uranate (Figure 5), which has a broad featureless spectrum with maximum intensity at 546 nm (18 300 cm^–1^).^75^ Furthermore, PHREEQC modeling indicated that when the U(VI) loading was reduced from 1 to 0.1 wt %, the saturation index of compreignacite at pH 11.5 decreased and sodium uranate is predicted to be the most oversaturated phase (Figure S23). When the U(VI) loading was further reduced to 0.01 wt %, the model predicted that clarkeite (Na(UO_2_)O(OH)·H_2_O) and sodium uranate (Na_2_U_2_O_7_) were oversaturated at pH 11.5 but sodium compreignacite (Na_2_(UO_2_)_6_O_4_(OH)_6_·7H_2_O) was not (Figure S23). These trends in the modeling results mirror those observed in the EXAFS and luminescence results and confirmed the gradual transition from uranyl-like to uranate-like speciation as the U(VI) loading decreases from 1 to 0.01 wt % at pH 11.5.

The gradual transition from uranyl-like precipitates to uranate-like precipitates is a result of changes in the U/Na ratio. While higher U/Na ratios favor the formation of U(VI) uranyl oxyhydroxide precipitates like compreignacite, increasing the relative concentration of sodium disrupts the formation of the repeating U(VI) polyhedral units in the uranyl oxyhydroxide precipitate structure.^8,86,87,90^ In this case, the increase in uranate formation is due to a reduction in the total U concentration in the lower U loading experiments. As such, the structure of sodium uranate, which has a higher sodium content than compreignacite, becomes more stable.^8^ Similar uranium speciation changes have been observed in a study where lithium content was varied in U-doped borate glasses and where increased Li content (and thus decreased U/Li ratio) led to a more uranate-like coordination environment, further highlighting the influence of alkali cations on U(VI) phase stability.^91^

### Surface Complexation

Finally, the XAS, luminescence, and solution modeling for the pH 9 0.01 wt % and pH 7 1 wt % to 0.01 wt % samples suggested that under these experimental conditions, surface complexation was the dominant U(VI) sorption mechanism. At pH 9, the transition from uranyl surface precipitates to surface complexes when the U(VI) loading was reduced to 0.01 wt % U(VI) was supported by EXAFS fitting, luminescence data, and PHREEQC modeling (Table S5). For EXAFS, the best fit included 2 O backscatterers at 1.83 Å, a split equatorial shell of 2.5 O at 2.24 Å, and 3 O at 2.43 Å and a U–Mg shell at 3.29 Å (*n* = 1) that was *F*-tested as statistically relevant (Table S5). The inclusion of the U–Mg shell in the fit, combined with the absence of a U–U shell, was consistent with past work on U(VI) adsorption on LDHs and shows inner sphere adsorption of U(VI) to the colloidal hydrotalcite.^36^ The luminescence data for this sample consisted of well-resolved peaks located at 482 nm (20 747 cm^–1^), 502 nm (19 920 cm^–1^), 522 nm (19 157 cm^–1^), and 544 nm (18 382 cm^–1^), confirming the presence of adsorbed uranyl (Figure S5).^78,84^ The luminescence lifetime data were fitted with lifetimes of 137 ± 2 μs (21%) and 676 ± 22 μs (79%), again suggesting the presence of two emissive U(VI) species. The longer lifetime value presumably corresponds to a uranyl surface complex, with adsorption directly to the surface of the hydrotalcite reducing the number of carbonate/water ligand(s) surrounding the U(VI) center, thereby lengthening the lifetime. Similar lifetime values (580 ± 240 μs) have been observed in other studies for U(VI) sorbed onto mineral surfaces as inner-sphere surface complexes.^9,71^ PHREEQC modeling of the 0.01 wt % U(VI) pH 9 system showed uranyl and uranate precipitates were undersaturated (Figure S23), which suggests that U(VI) surface complexes are more significant than uranyl surface precipitates under these conditions.

For the pH 7 1, 0.1, and 0.01 wt % U(VI) samples, the EXAFS best fits were all similar and included 2 O backscatterers at 1.81–1.82 Å (Tables S3–S5). For the 1 and 0.1 wt % U(VI) systems, between 5 and 5.5 O backscatterers were modeled in a single-shell environment at 2.45–2.46 Å (Tables S3 and S4). For the 0.01 wt % U(VI) sample, a total of 6 O backscatterers were fit in a split shell with 1.5 O at 2.29 Å and 4.5 O at 2.46 Å (Table S5). At pH 7, U–C shells were present in all three fits at 2.90(2) Å, with the U–C coordination number systematically decreasing from 3 to 2.2 as the U loading decreased from 1 to 0.01 wt % U(VI). For all pH 7 samples, the distant peak apparent in the Fourier transform was best modeled using a U–O_dis_ shell between 4.09 and 4.15 Å, with the coordination number decreasing from 2 to 1 as the U(VI) loading was reduced from 1 to 0.01 wt %.^70^ U–Mg shells (*n* = 1.3–1.5) between 3.33 and 3.35 Å were included in the fits for the pH 7 0.1 and 0.01 wt % samples after *F*-testing indicated that their inclusion was statistically relevant (Table S5).

Overall, at pH 7, the EXAFS fits are consistent with U(VI) triscarbonate, suggesting a change in the speciation of sorbed U(VI) under circumneutral conditions when compared to pH 11.5 where surface precipitates dominate.^70,92^ Interestingly, the absence of a U–Mg shell in the 1 wt % fit suggested that the U(VI) triscarbonato species may be largely outer-sphere sorbed to the hydrotalcite nanoparticles, similar to past work investigating U(VI) sorption onto brucite and magnesite.^45^ As the U(VI) loading fell to 0.1 and 0.01 wt % U(VI), the inclusion of a statistically relevant U–Mg shell at 3.33–3.35 Å in the fits suggested that inner-sphere complexes were present and becoming increasingly significant as the U(VI) loading was reduced (Table S4). These U–Mg shell distances are within the range published by Gräfe et al., who postulated that the U–Mg distances for inner-sphere U(VI) carbonate surface complexes on calcined hydrotalcite precipitates could vary between 3.1 and 3.9 Å depending on the number of carbonate ligands also present in the equatorial plane.^38^ More specifically, the distance of the U–Mg shells (3.33–3.35 Å) included in the pH 7 0.1 and 0.01 wt % fits in this study agree with the sorption mode postulated by Gräfe et al., where U(VI) is binding to the edge sites of the hydrotalcite nanoparticle via two equatorial oxygens to form mononuclear, bidentate U(VI) inner-sphere surface complexes.^38^ Evidence for this bidentate, inner-sphere sorption mode is seen in the pH 7 0.01 wt % fit, with the splitting in the oxygen equatorial shell caused by the presence of shorter U–O_eq_ distances which form as a result of the direct binding of the U(VI) carbonate species as an inner-sphere complex to the hydrotalcite surface (Table S5). A similar U–O_eq_ shell splitting was observed by Elzinga et al. when studying U(VI) sorption to calcite, with the author also finding that inner-sphere complexation became more significant as the U(VI) loading was reduced.^71^ Splitting in the U–O_eq_ shell is not observed for the two higher pH 7 U(VI) loading samples, indicating that outer-sphere U(VI) complexes were more significant due to the edge sites becoming saturated as the U(VI) loading was increased.

The luminescence spectra for both the pH 7 U(VI) 1 and 0.01 wt % samples in Figure 5 showed six well-resolved features. For the 1 wt % sample, the vibrational progression is located at 482 nm (20 747 cm^–1^), 502 nm (19 920 cm^–1^), 522 nm (19 157 cm^–1^), 548 nm (18 248 cm^–1^), 570 nm (17 544 cm^–1^), and 598 nm (16 722 cm^–1^), while for the 0.01 wt %, the six features were red-shifted by approximately 4 nm. Both spectra are consistent with published data for both UO_2_(CO_3_)_3_^4–^_(aq)_ and ternary magnesium uranyl carbonate complexes.^71,84,92^ The luminescence results are again in agreement with the EXAFS modeling, which indicated that U(VI) triscarbonate dominated at pH 7. Luminescence analysis of the pH 7 samples yielded lifetime fits with biexponential decay kinetics, consistent with two distinct U(VI) binding environments. For the 1 wt % sample, the lifetime values are 1257 ± 10 μs (76%) and 688 ± 15 μs (24%), while for the 0.01 wt % sample, the values are 1189 ± 7 μs (82%) and 493 ± 8 μs (18%). Here, for both the pH 7 1 and 0.01 wt % samples, the longer lifetimes were due to poorly quenched U(VI) inner-sphere complexes, while the shorter fluorescence lifetimes were attributed to outer sphere complexation, where U(VI) carbonate ligands and the associated hydration sphere enhancing quenching. Indeed, the simultaneous adsorption of inner- and outer-sphere U(VI) carbonate surface complexes has been observed on other mineral surfaces.^93^ Additionally, the 4 nm red shift between the spectral features of the 0.01 wt % sample relative to the 1 wt % sample indicates changes in the relative proportions of the outer- and inner-sphere complexes, with inner-sphere complexes becoming more significant as the U(VI) loading is reduced.^86^ A similar shift was observed by Jo et al., who found that the spectrum for U(VI) inner-sphere sorbed onto the surface of γ-alumina was red-shifted by approximately 4 nm when compared to that of an aqueous Ca triscarbonato species.^76^

When considering changes in the U(VI) aqueous speciation between pH 7 and 11.5, PHREEQC modeling suggested that below pH 9.5, the UO_2_(CO_3_)_3_^4–^_(aq)_ and Mg(UO_2_(CO_3_)_3_)^2–^_(aq)_ species dominated, with Mg(UO_2_(CO_3_)_3_)^2–^_(aq)_ becoming more significant at pH <8 due to Mg^2+^ leaching from the hydrotalcite (Figures S21–S23), thus resulting in an increased dissolved Mg_(aq)_^2+^ concentration. This agrees with the EXAFS and luminescence data which indicated that the U(VI) triscarbonato speciation was dominant at pH 7, regardless of the U(VI) loading. Similar alkaline earth U(VI) triscarbonato complexes are significant in environmental scenarios and have a range of potential counterions, including Ca^2+^, Na^+^, and Sr^2+^.^59^ Interestingly, the soluble Ca analogue of this species, Ca(UO_2_(CO_3_)_3_)^2–^_(aq)_, as well as Ca_2_(UO_2_(CO_3_)_3_)^0^_(aq)_, is reportedly significant in calcite–U(VI) systems under circumneutral conditions.^9,71^ Indeed, it was reported that the presence of these aqueous species prevented solid U(VI) precipitation onto the surface of the calcite, with monomeric surface complexes forming preferentially.^71,94,95^ Other studies have also reported that the formation of alkaline earth uranyl triscarbonate species inhibits solid U(VI) precipitation onto various mineral surfaces.^59,96,97^ The results in this study indicate that similar changes in U(VI) sorption behavior occur in the hydrotalcite–U(VI) systems under more circumneutral conditions, with Mg leaching and the exchange of interlayer carbonate resulting in the formation of U(VI) carbonate species such as Mg(UO_2_(CO_3_)_3_)^2–^_(aq)_ and UO_2_(CO_3_)_3_^4–^_(aq)_. The presence of these anionic species then favors the formation of monomeric surface complexes rather than U(VI) surface precipitates. Again, PHREEQC modeling supported these observations as in 1, 0.1, and 0.01 wt % experiments, no U(VI) phases are oversaturated at pH 7 (Figures S21–S23), and Mg(UO_2_(CO_3_)_3_)^2–^ and UO_2_(CO_3_)_3_^4–^ aqueous complexes are expected to dominate. Here, the XAS, luminescence, and ultrafiltration data all confirm the elevated reactivity of these aqueous species to the hydrotalcite colloid through the formation of both inner- and/or outer-sphere complexes at pH 7 and at low loading at pH 9.

**Figure 6:**
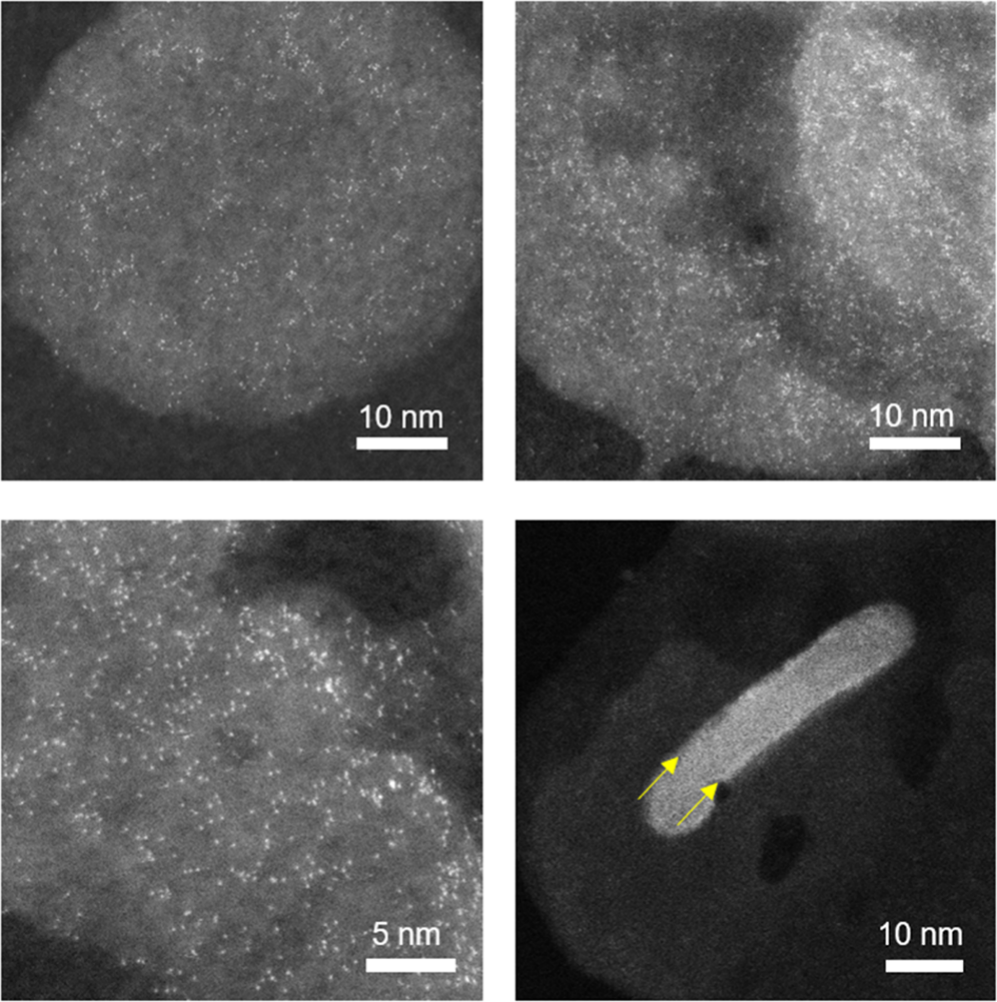
HAADF STEM images of hydrotalcite nanoparticles. The nanoparticles were collected from a colloidal system with a U(VI) loading of 1 wt % and at pH 7.

High-resolution HAADF STEM images of hydrotalcite nanoparticles taken from a pH 7 1 wt % U(VI) colloidal hydrotalcite system show high-intensity spots distributed across the hydrotalcite nanoparticles (Figure 6). Given the strong dependency of this imaging mode on atomic number and the small size of these features (<2 Å), these bright spots can be identified as uranium atomic species or complexes containing an individual uranium atom or ion. Cross-sectional imaging of the hydrotalcite nanoparticles confirms that the uranium is concentrated on the surfaces of the hydrotalcite nanoparticles (highlighted by the yellow arrows in Figure 6 (bottom right) and in Figure S24). Furthermore, the highly dispersed nature of the U(VI) spot features suggests that these are present as individual adsorbed surface complexes rather than discrete U(VI) surface precipitates (Figures 6 and S24). This agrees with the above EXAFS, luminescence, and solution modeling, all of which suggest that at pH 7, U(VI) carbonate surface complexes form rather than U(VI) surface precipitates.^71,98^ The presence of U(VI) complexes on the surface of the hydrotalcite nanoparticles is also in agreement with both the EXAFS results, which suggest that at a U(VI) loading of 1 wt %, outer-sphere surface complexes dominate, and also with the results in past work which evidenced the formation of U(VI) outer-sphere surface complexes on Mg-based mineral surfaces under circumneutral conditions.^45^

## Conclusions

This study has shown that hydrotalcite forms stable colloids over several weeks between pH 7 and 11.5. The colloidal stability was comparatively low at pH 11.5 as the positive charge on the hydrotalcite nanoparticles was reduced, causing flocculation. This suggests that maintaining elevated pH conditions (pH ≥ 11) in solution will favor hydrotalcite (and associated sorbed radionuclides) being partitioned to solids. Between pH 8–7, Mg^2+^ leached from hydrotalcite, resulting in a gibbsite-like nanoparticle surface composition, which again promoted flocculation. U(VI) sorbed to hydrotalcite between pH 7 and 11.5 (greater than 98.5% sorption), regardless of pH, U(VI) loading, or time. Increased U(VI) loadings enhanced the flocculation of the colloidal hydrotalcite, presumably due to U(VI) sorption to the nanoparticle surfaces causing a reduction in the overall positive charge. The U(VI) sorption to hydrotalcite was affected by pH and U(VI) concentration. At pH 11.5 and 1 wt % U(VI), U(VI)–oxyhydroxide surface precipitates formed, and these precipitates became more uranate-like as the U(VI) loading fell to 0.1 and 0.01 wt %. At pH 9, U(VI)–oxyhydroxide surface precipitates formed at 1 and 0.1 wt % U(VI), while U(VI) surface complexes became more significant at 0.01 wt % U(VI). Under circumneutral conditions, enhanced exchange of interlayer carbonate and leaching of Mg^2+^ to the aqueous phase led to the formation of Mg(UO_2_(CO_3_)_3_)^2–^ and UO_2_(CO_3_)_3_^4–^ surface complexes with the hydrotalcite, with inner sphere complexes favored at lower U(VI) loadings.

The results of this study are directly relevant to spent nuclear fuel storage facilities. Furthermore, the behavior of U(VI) as UO_2_^2+^ also provides insight into plutonium (as PuO_2_^2+^, a radionuclide which is pertinent in spent fuel storage ponds) behavior. Here, Pu(VI)O_2_^2+^ ions present in a fuel pond environment under oxic and carbonated conditions will result in the Pu(VI)O_2_^2+^ partitioning to hydrotalcite. In addition, the improved mechanistic understanding of U(VI) sorption to colloidal hydrotalcite under variable pH and U(VI) concentrations can be used to inform and predict U(VI) behavior across a range of environmental scenarios where LDHs or colloidal particles are present. Such scenarios include the clean-up and remediation of regions such as the Hanford site, USA, and Fukushima, Japan, where colloid-facilitated radionuclide transport has been confirmed,^99−101^ the efficient extraction of U from waters,^102,103^ or in cementitious geological disposal facilities, where hydrotalcite is predicted to form.^104,105^

## Supporting Information Available

Complete ultrafiltration dataset, settling experiment data, XRD results, FTIR spectra and further mechanistic detail of U(VI) sorption to hydrotalcite is provided in the form of tables containing EXAFS fitting parameters, XANES comparison plots, PHREEQC geochemical modeling plots, and TEM images (PDF)

## References

[ref1] IAEA. IAEA Nuclear Energy Series: Status and Trends in Spent Fuel and Radioactive Waste Management; IAEA Nuclear Energy Series, 2018.

[ref2] Manon B.; Marcos B.; IanFairlie G. The World Nuclear Waste Report 2019, 2019; pp 1–147.

[ref3] Salbu B.; Krekling T.; Oughton D. H.; Østby G.; Kashparov V. A.; Brand T. L.; Day J. P. Hot Particles in Accidental Releases from Chernobyl and Windscale Nuclear Installations. Analyst 1994, 119, 125–130. 10.1039/an9941900125.

[ref4] Falck W. E. Radioactive and Other Environmental Contamination from Uranium Mining and Milling. In Environmental Remediation and Restoration of Contaminated Nuclear and Norm Sites; Elsevier, 2015; pp 3–34.

[ref5] Clark D. L.; Hobart D. E.; Neu M. P. Actinide Carbonate Complexes and Their Importance in Actinide Environmental Chemistry. Chem. Rev. 1995, 95, 25–48. 10.1021/cr00033a002.

[ref6] Gorman-Lewis D.; Fein J. B.; Burns P. C.; Szymanowski J. E. S.; Converse J. Solubility Measurements of the Uranyl Oxide Hydrate Phases Metaschoepite, Compreignacite, Na-Compreignacite, Becquerelite, and Clarkeite. J. Chem. Thermodyn. 2008, 40, 980–990. 10.1016/j.jct.2008.02.006.

[ref7] Kenney J. P. L.; Kirby M. E.; Cuadros J.; Weiss D. J. A Conceptual Model to Predict Uranium Removal from Aqueous Solutions in Water-Rock Systems Associated with Low- and Intermediate-Level Radioactive Waste Disposal. RSC Adv. 2017, 7, 7876–7884. 10.1039/c6ra26773d.

[ref8] Ding W. Syntheses of Ternary Oxyhydrates and Oxides in the Calcium- Uranium System. Ph.D. Thesis, The University of Leeds, 2017.

[ref9] Smith K. F.; Bryan N. D.; Swinburne A. N.; Bots P.; Shaw S.; Natrajan L. S.; Mosselmans J. F. W.; Livens F. R.; Morris K. U(VI) Behaviour in Hyperalkaline Calcite Systems. Geochim. Cosmochim. Acta 2015, 148, 343–359. 10.1016/j.gca.2014.09.043.

[ref10] Bots P.; Morris K.; Hibberd R.; Law G. T. W.; Mosselmans J. F. W.; Brown A. P.; Doutch J.; Smith A. J.; Shaw S. Formation of Stable Uranium(VI) Colloidal Nanoparticles in Conditions Relevant to Radioactive Waste Disposal. Langmuir 2014, 30, 14396–14405. 10.1021/la502832j. 25418066

[ref11] Matsunaga T.; Nagao S.; Ueno T.; Takeda S.; Amano H.; Tkachenko Y. Association of Dissolved Radionuclides Released by the Chernobyl Accident with Colloidal Materials in Surface Water. Appl. Geochem. 2004, 19, 1581–1599. 10.1016/j.apgeochem.2004.02.002.

[ref12] Kersting A. B. Plutonium Transport in the Environment. Inorg. Chem. 2013, 52, 3533–3546. 10.1021/ic3018908. 23458827

[ref13] Parry S. A.; O’Brien L.; Fellerman A. S.; Eaves C. J.; Milestone N. B.; Bryan N. D.; Livens F. R. Plutonium Behaviour in Nuclear Fuel Storage Pond Effluents. Energy Environ. Sci. 2011, 4, 1457–1464. 10.1039/c0ee00390e.

[ref14] Maher Z.; Ivanov P.; O’Brien L.; Sims H.; Taylor R. J.; Heath S. L.; Livens F. R.; Goddard D.; Kellet S.; Rand P.; Bryan N. D. Americium and Plutonium Association with Magnesium Hydroxide Colloids in Alkaline Nuclear Industry Process Environments. J. Nucl. Mater. 2016, 468, 84–96. 10.1016/j.jnucmat.2015.11.010.

[ref15] Wilson P. D. The Nuclear Fuel Cycle—From Ore to Waste; Oxford University Press, 1997.

[ref16] Jackson S. F.; Monk S. D.; Riaz Z. An Investigation towards Real Time Dose Rate Monitoring, and Fuel Rod Detection in a First Generation Magnox Storage Pond (FGMSP). Appl. Radiat. Isot. 2014, 94, 254–259. 10.1016/j.apradiso.2014.08.019. 25244071

[ref17] Foster L.; Boothman C.; Ruiz-Lopez S.; Boshoff G.; Jenkinson P.; Sigee D.; Pittman J. K.; Morris K.; Lloyd J. R. Microbial Bloom Formation in a High PH Spent Nuclear Fuel Pond. Sci. Total Environ. 2020, 720, 137515 10.1016/j.scitotenv.2020.137515. 32325573

[ref18] The Magnox Operating Programme 9 (MOP 9); Nuclear Decommissioning Authority, 2012; pp 1–13.

[ref19] Alpha Guidelines (Abridged); Sellafield Ltd., 2017; pp 1–20.

[ref20] Oxide Fuels - Preferred Option; Nuclear Decommissioning Authority, 2012; pp 1–21.

[ref21] Gregson C. R.; Hastings J. J.; Sims H. E.; Steele H. M.; Taylor R. J. Characterisation of Plutonium Species in Alkaline Liquors Sampled from a UK Legacy Nuclear Fuel Storage Pond. Anal. Methods 2011, 3, 1957–1968. 10.1039/c1ay05313b.

[ref22] Gregson C. R.; Goddard D. T.; Sarsfield M. J.; Taylor R. J. Combined Electron Microscopy and Vibrational Spectroscopy Study of Corroded Magnox Sludge from a Legacy Spent Nuclear Fuel Storage Pond. J. Nucl. Mater. 2011, 412, 145–156. 10.1016/j.jnucmat.2011.02.046.

[ref23] van Veelen A.; Copping R.; Law G. T. W.; Smith A. J.; Bargar J. R.; Rogers J.; Shuh D. K.; Wogelius R. A. Uranium Uptake onto Magnox Sludge Minerals Studied Using EXAFS. Mineral. Mag. 2012, 76, 3095–3104. 10.1180/minmag.2012.076.8.24.

[ref24] Bochkarev G. R.; Pushkareva G. I. Strontium Removal from Aqueous Media by Natural and Modified Sorbents. J. Min. Sci. 2009, 45, 290–294. 10.1007/s10913-009-0036-3.

[ref25] Yu W.; Du N.; Gu Y.; Yan J.; Hou W. Specific Ion Effects on the Colloidal Stability of Layered Double Hydroxide Single-Layer Nanosheets. Langmuir 2020, 36, 6557–6568. 10.1021/acs.langmuir.0c01089. 32466650

[ref26] Gassin P.-M.; Prelot B.; Grégoire B.; Martin-Gassin G. Second-Harmonic Scattering in Layered Double Hydroxide Colloids: A Microscopic View of Adsorption and Intercalation. Langmuir 2018, 34, 12206–12213. 10.1021/acs.langmuir.8b02161. 30203976

[ref27] Layrac G.; Destarac M.; Gérardin C.; Tichit D. Highly Stable Layered Double Hydroxide Colloids: A Direct Aqueous Synthesis Route from Hybrid Polyion Complex Micelles. Langmuir 2014, 30, 9663–9671. 10.1021/la502159x. 25087853

[ref28] Zhang J.; Luan L.; Zhu W.; Liu S.; Sun D. Phase Behavior of Aqueous Suspensions of Mg2Al Layered Double Hydroxide: The Competition among Nematic Ordering, Sedimentation, and Gelation. Langmuir 2007, 23, 5331–5337. 10.1021/la0625300. 17439162

[ref29] Wypych F., Satyanarayana K. G., Eds. In Clay Surfaces—Fundamentals and Applications; Elsevier, 2004.

[ref30] Douglas G.; Shackleton M.; Woods P. Hydrotalcite Formation Facilitates Effective Contaminant and Radionuclide Removal from Acidic Uranium Mine Barren Lixiviant. Appl. Geochem. 2014, 42, 27–37. 10.1016/j.apgeochem.2013.12.018.

[ref31] Mattigod S. V.; Fryxell G. E.; Serne R. J.; Parker K. E. Evaluation of Novel Getters for Adsorption of Radioiodine from Groundwater and Waste Glass Leachates. Radiochim. Acta 2003, 91, 539–546. 10.1524/ract.91.9.539.20001.

[ref32] Li D.; Kaplan D. I. Sorption Coefficients and Molecular Mechanisms of Pu, U, Np, Am and Tc to Fe (Hydr)Oxides: A Review. J. Hazard. Mater. 2012, 243, 1–18. 10.1016/j.jhazmat.2012.09.011. 23141377

[ref33] Kremleva A.; Krüger S.; Rösch N. Uranyl Adsorption at Solvated Edge Surfaces of 2-1 Smectites. A Density Functional Study. Phys. Chem. Chem. Phys. 2015, 17, 13757–13768. 10.1039/c5cp01074h. 25941904

[ref34] Schindler M.; Hawthorne F. C.; Putnis C.; Putnis A. Growth of Uranyl-Hydroxy-Hydrate and Uranyl-Carbonate Minerals on the (104) Surface of Calcite. Can. Mineral. 2004, 42, 1683–1697. 10.2113/gscanmin.42.6.1683.

[ref35] Marshall T. A.; Morris K.; Law G. T. W.; Livens F. R.; Mosselmans J. F. W.; Bots P.; Shaw S. Incorporation of Uranium into Hematite during Crystallization from Ferrihydrite. Environ. Sci. Technol. 2014, 48, 3724–3731. 10.1021/es500212a. 24580024 PMC4059770

[ref36] Roberts H. E.; Morris K.; Law G. T. W.; Mosselmans J. F. W.; Bots P.; Kvashnina K.; Shaw S. Uranium(V) Incorporation Mechanisms and Stability in Fe(II)/Fe(III) (Oxyhydr)Oxides. Environ. Sci. Technol. Lett. 2017, 4, 421–426. 10.1021/acs.estlett.7b00348.

[ref37] Ma S.; Huang L.; Ma L.; Shim Y.; Islam S. M.; Wang P.; Zhao L.-D.; Wang S.; Sun G.; Yang X.; Kanatzidis M. G. Efficient Uranium Capture by Polysulfide/Layered Double Hydroxide Composites. J. Am. Chem. Soc. 2015, 137, 3670–3677. 10.1021/jacs.5b00762. 25714654

[ref38] Gräfe M.; Bunney K. G.; Cumberland S.; Douglas G. Mechanisms of Uranyl Sequestration by Hydrotalcite. ACS Omega 2017, 2, 7112–7119. 10.1021/acsomega.7b01050. 31457291 PMC6645089

[ref39] Xu Z. P.; Jin Y.; Liu S.; Hao Z. P.; Lu G. Q. Max Surface Charging of Layered Double Hydroxides during Dynamic Interactions of Anions at the Interfaces. J. Colloid Interface Sci. 2008, 326, 522–529. 10.1016/j.jcis.2008.06.062. 18674775

[ref40] Kulyukhin S. A.; Krasavina E. P.; Gredina I. V.; Mizina L. V. Sorption of U(VI) from Aqueous Solutions on Layered Double Hydroxides of Mg, Al, and Nd. Radiochemistry 2010, 52, 653–661. 10.1134/s1066362210060160.

[ref41] Zhang H.; Wang J.; Zhang B.; Liu Q.; Li S.; Yan H.; Liu L. Synthesis of a Hydrotalcite-like Compound from Oil Shale Ash and Its Application in Uranium Removal. Colloids Surf., A 2014, 444, 129–137. 10.1016/j.colsurfa.2013.12.054.

[ref42] Tu J.; Peng X.; Wang S.; Tian C.; Deng H.; Dang Z.; Lu G.; Shi Z.; Lin Z. Effective Capture of Aqueous Uranium from Saline Lake with Magnesium-Based Binary and Ternary Layered Double Hydroxides. Sci. Total Environ. 2019, 677, 556–563. 10.1016/j.scitotenv.2019.04.429. 31063897

[ref43] Yao W.; Wang X.; Liang Y.; Yu S.; Gu P.; Sun Y.; Xu C.; Chen J.; Hayat T.; Alsaedi A.; Wang X. Synthesis of Novel Flower-like Layered Double Oxides/Carbon Dots Nanocomposites for U(VI) and 241Am(III) Efficient Removal: Batch and EXAFS Studies. Chem. Eng. J. 2018, 332, 775–786. 10.1016/j.cej.2017.09.011.

[ref44] Song S.; Yin L.; Wang X.; Liu L.; Huang S.; Zhang R.; Wen T.; Yu S.; Fu D.; Hayat T.; Wang X. Interaction of U(VI) with Ternary Layered Double Hydroxides by Combined Batch Experiments and Spectroscopy Study. Chem. Eng. J. 2018, 338, 579–590. 10.1016/j.cej.2018.01.055.

[ref45] Van Veelen A.; Bargar J. R.; Law G. T. W.; Brown G. E.; Wogelius R. A. Uranium Immobilization and Nanofilm Formation on Magnesium-Rich Minerals. Environ. Sci. Technol. 2016, 50, 3435–3443. 10.1021/acs.est.5b06041. 26990311

[ref46] Douglas G. B.; Wendling L. A.; Pleysier R.; Trefry M. G. Hydrotalcite Formation for Contaminant Removal from Ranger Mine Process Water. Mine Water Environ. 2010, 29, 108–115. 10.1007/s10230-010-0106-4.

[ref47] Kauffman G. B. The Chemistry of the Actinide and Transactinide Elements, 3rd ed.; Morss L. R., Edelstein L. R., Fuger J., Katz J. J., Eds.; Springer, 2007; Vol. 5.

[ref48] Schmidt M.; Wilson R. E.; Lee S. S.; Soderholm L.; Fenter P. Adsorption of Plutonium Oxide Nanoparticles. Langmuir 2012, 28, 2620–2627. 10.1021/la2037247. 22216888

[ref49] Xu Z. P.; Stevenson G.; Lu C.-Q.; Lu G. Q. Dispersion and Size Control of Layered Double Hydroxide Nanoparticles in Aqueous Solutions. J. Phys. Chem. B 2006, 110, 16923–16929. 10.1021/jp062281o. 16927982

[ref50] Weatherill J. S. Iron Oxyhydroxide Formation in the Enhanced Actinide Removal Plant. Ph.D. Thesis, University of Manchester, 2017.

[ref51] Neill T. S.; Morris K.; Pearce C. I.; Sherriff N. K.; Burke M. G.; Chater P. A.; Janssen A.; Natrajan L.; Shaw S. Stability, Composition, and Core-Shell Particle Structure of Uranium(IV)-Silicate Colloids. Environ. Sci. Technol. 2018, 52, 9118–9127. 10.1021/acs.est.8b01756. 30001122

[ref52] Dreissig I.; Weiss S.; Hennig C.; Bernhard G.; Zänker H. Formation of Uranium(IV)-Silica Colloids at near-Neutral PH. Geochim. Cosmochim. Acta 2011, 75, 352–367. 10.1016/j.gca.2010.10.011.

[ref53] Laurent T. C.; Granath K. A. Fractionation of Dextran and Ficoll by Chromatography on Sephadex G-200. Biochim. Biophys. Acta 1967, 136, 191–198. 10.1016/0304-4165(67)90063-3. 6049492

[ref54] Neill T. S.; Morris K.; Pearce C. I.; Abrahamsen-Mills L.; Kovarik L.; Kellet S.; Rigby B.; Vitova T.; Schacherl B.; Shaw S. Silicate Stabilisation of Colloidal UO2 Produced by Uranium Metal Corrosion. J. Nucl. Mater. 2019, 526, 151751 10.1016/j.jnucmat.2019.151751.

[ref55] Dent A. J.; Cibin G.; Ramos S.; Smith A. D.; Scott S. M.; Varandas L.; Pearson M. R.; Krumpa N. A.; Jones C. P.; Robbins P. E. B18: A Core XAS Spectroscopy Beamline for Diamond. J. Phys. Conf. 2009, 190, 012039 10.1088/1742-6596/190/1/012039.

[ref56] Ravel B.; Newville M. ATHENA,ARTEMIS,HEPHAESTUS: data analysis for X-ray absorption spectroscopy usingIFEFFIT. J. Synchrotron Radiat. 2005, 12, 537–541. 10.1107/s0909049505012719. 15968136

[ref57] Jones D. L.; Andrews M. B.; Swinburne A. N.; Botchway S. W.; Ward A. D.; Lloyd J. R.; Natrajan L. S. Fluorescence Spectroscopy and Microscopy as Tools for Monitoring Redox Transformations of Uranium in Biological Systems. Chem. Sci. 2015, 6, 5133–5138. 10.1039/c5sc00661a. 29142731 PMC5666681

[ref58] Parkhurst D. L.; Appelo C. A. J. PHREEQC (Version 3)-A Computer Program for Speciation, Batch-Reaction, One-Dimensional Transport, and Inverse Geochemical Calculations; U.S. Geological Survey, 1999.

[ref59] Dong W.; Brooks S. C. Determination of the Formation Constants of Ternary Complexes of Uranyl and Carbonate with Alkaline Earth Metals (Mg2+, Ca2+, Sr 2+, and Ba2+) Using Anion Exchange Method. Environ. Sci. Technol. 2006, 40, 4689–4695. 10.1021/es0606327. 16913125

[ref60] Wiyantoko B.; Kurniawati P.; Purbaningtias T. E.; Fatimah I. Synthesis and Characterization of Hydrotalcite at Different Mg/Al Molar Ratios. Procedia Chem. 2015, 17, 21–26. 10.1016/j.proche.2015.12.115.

[ref61] Omonmhenle S. I.; Shannon I. J. Synthesis and Characterisation of Surfactant Enhanced Mg-Al Hydrotalcite-like Compounds as Potential 2-Chlorophenol Scavengers. Appl. Clay Sci. 2016, 127–128, 88–94. 10.1016/j.clay.2016.03.033.

[ref62] Liu Y.; Ma D.; Blackley R. A.; Zhou W.; Han X.; Bao X. Synthesis and Characterization of Gibbsite Nanostructures. J. Phys. Chem. C 2008, 112, 4124–4128. 10.1021/jp7101572.

[ref63] Iyi N.; Matsumoto T.; Kaneko Y.; Kitamura K. Deintercalation of Carbonate Ions from a Hydrotalcite-like Compound: Enhanced Decarbonation Using Acid-Salt Mixed Solution. Chem. Mater. 2004, 16, 2926–2932. 10.1021/cm049579g.

[ref64] Iyi N.; Sasaki T. Decarbonation of MgAl-LDHs (Layered Double Hydroxides) Using Acetate-Buffer/NaCl Mixed Solution. J. Colloid Interface Sci. 2008, 322, 237–245. 10.1016/j.jcis.2008.02.047. 18377921

[ref65] Iyi N.; Yamada H. One-Step Conversion of CO32- LDH (Layered Double Hydroxide) into Anion-Exchangeable LDHs Using an Acetate-Buffer/Salt Method. Chem. Lett. 2010, 39, 591–593. 10.1246/cl.2010.591.

[ref66] Labajos F. M.; Rives V.; Ulibarri M. A. Effect of Hydrothermal and Thermal Treatments on the Physicochemical Properties of Mg-Al Hydrotalcite-like Materials. J. Mater. Sci. 1992, 27, 1546–1552. 10.1007/bf00542916.

[ref67] Hibino T. Anion Selectivity of Layered Double Hydroxides: Effects of Crystallinity and Charge Density. Eur. J. Inorg. Chem. 2018, 722–730. 10.1002/ejic.201701067.

[ref68] Jobbágy M.; Regazzoni A. E. Dissolution of Nano-Size Mg-Al-Cl Hydrotalcite in Aqueous Media. Appl. Clay Sci. 2011, 51, 366–369. 10.1016/j.clay.2010.11.027.

[ref69] Kosmulski M. The PH-Dependent Surface Charging and the Points of Zero Charge. J. Colloid Interface Sci. 2002, 253, 77–87. 10.1006/jcis.2002.8490. 16290833

[ref70] Catalano J. G.; Brown G. E. Analysis of Uranyl-Bearing Phases by EXAFS Spectroscopy: Interferences, Multiple Scattering, Accuracy of Structural Parameters, and Spectral Differences. Am. Mineral. 2004, 89, 1004–1021. 10.2138/am-2004-0711.

[ref71] Elzinga E. J.; Tait C. D.; Reeder R. J.; Rector K. D.; Donohoe R. J.; Morris D. E. Spectroscopic Investigation of U(VI) Sorption at the Calcite-Water Interface. Geochim. Cosmochim. Acta 2004, 68, 2437–2448. 10.1016/j.gca.2003.09.023.

[ref72] Burns P. C. The Structure of Compreignacite, K2[(UO2)3O2(OH)3] 2(H2O)7. Can. Mineral. 1998, 36, 1061–1067.

[ref73] Burns P. C.; Ewing R. C.; Miller M. L. Incorporation Mechanisms of Actinide Elements into the Structures of U6+ Phases Formed during the Oxidation of Spent Nuclear Fuel. J. Nucl. Mater. 1997, 245, 1–9. 10.1016/s0022-3115(97)00006-8.

[ref74] Arnold T.; Baumann N. Boltwoodite [K(UO2)(SiO3OH)(H2O)1.5] and Compreignacite K2[(UO2)3O2(OH)3]2·7H2O Characterized by Laser Fluorescence Spectroscopy. Spectrochim. Acta, Part A 2009, 71, 1964–1968. 10.1016/j.saa.2008.07.029. 18789751

[ref75] Tits J.; Geipel G.; Macé N.; Eilzer M.; Wieland E. Determination of Uranium(VI) Sorbed Species in Calcium Silicate Hydrate Phases: A Laser-Induced Luminescence Spectroscopy and Batch Sorption Study. J. Colloid Interface Sci. 2011, 359, 248–256. 10.1016/j.jcis.2011.03.046. 21489548

[ref76] Jo Y.; Lee J.-Y.; Yun J.-I. Adsorption of Uranyl Tricarbonate and Calcium Uranyl Carbonate onto γ-Alumina. Appl. Geochem. 2018, 94, 28–34. 10.1016/j.apgeochem.2018.05.004.

[ref77] Amayri S.; Arnold T.; Foerstendorf H.; Geipel G.; Bernhard G. Spectroscopic Characterization of Synthetic Becquerelite, Ca[(UO2)6O4(OH)6]·8H 2O, and Swartzite,CaMg[UO2(CO3) 3]·12H2O. Can. Mineral. 2004, 42, 953–962. 10.2113/gscanmin.42.4.953.

[ref78] De Hair J. T. W.; Blasse G. Luminescence of the Octahedral Uranate Group. J. Lumin. 1976, 14, 307–323. 10.1016/0022-2313(76)90001-6.

[ref79] Blasse G. The Nature of the Luminescent Centres in Calcium Uranate (Ca3UO6). Solid State Commun. 1976, 19, 779–781. 10.1016/0038-1098(76)90917-0.

[ref80] Smith K. F. Radionuclide Behaviour in Hyperalkaline Systems Relevant to Geological Disposal of Radioactive Waste. Ph.D. Thesis; Univeristy of Manchester, 2014.

[ref81] Wang Z.; Zachara J. M.; Liu C.; Gassman P. L.; Felmy A. R.; Clark S. B. A Cryogenic Fluorescence Spectroscopic Study of Uranyl Carbonate, Phosphate and Oxyhydroxide Minerals. Radiochim. Acta 2008, 96, 591–598. 10.1524/ract.2008.1541.

[ref82] Gaft M.; Reisfeld R.; Panczer G. Modern Luminescence Spectroscopy of Minerals and Materials; Springer, 2005.

[ref83] Allen P. G.; Shuh D. K.; Bucher J. J.; Edelstein Ν. M.; Palmer C. E. A.; Silva R. J.; Nguyen S. N.; Marquez L. N.; Hudson E. A. Determinations of Uranium Structures by EXAFS: Schoepite and Other U(VI) Oxide Precipitates. Radiochim. Acta 1996, 75, 47–54. 10.1524/ract.1996.75.1.47.

[ref84] Philipp T.; Shams Aldin Azzam S.; Rossberg A.; Huittinen N.; Schmeide K.; Stumpf T. U(VI)Sorption on Ca-Bentonite at (Hyper)Alkaline Conditions – Spectroscopic Investigations of Retention Mechanisms. Sci. Total Environ. 2019, 676, 469–481. 10.1016/j.scitotenv.2019.04.274. 31048176

[ref85] De Hair J. T. W.; Blasse G. The Luminescence Properties of the Octahedral Uranate Group in Oxides with Perovskite Structure. J. Solid State Chem. 1976, 19, 263–270. 10.1016/0022-4596(76)90176-6.

[ref86] Hoekstra H.; Siegel S. Structural Studies on Li4UO5 and Na4UO5. J. Inorg. Nucl. Chem. 1964, 26, 693–700. 10.1016/0022-1902(64)80311-0.

[ref87] Bickel M.; Kanellakopulos B.; Powietzka B. The Structural and Electronic Properties of Na4UO5 and Na4NpO5. J. Less Common Met. 1991, 170, 161–169. 10.1016/0022-5088(91)90061-8.

[ref88] Read C. M.; Bugaris D. E.; Zur Loye H.-C. Single Crystal Growth and Structural Characterization of Four Complex Uranium Oxides: CaUO4, β-Ca3UO6, K 4CaU3O12, and K4SrU 3O12. Solid State Sci. 2013, 17, 40–45. 10.1016/j.solidstatesciences.2012.12.013.

[ref89] Downward L.; Booth C. H.; Lukens W. W.; Bridges F. A Variation of the F-Test for Determining Statistical Relevance of Particular Parameters in EXAFS Fits. AIP Conf. Proc. 2007, 882, 129 10.1063/1.2644450.

[ref90] Rietveld H. M. The Crystal Structure of Some Alkaline Earth Metal Uranates of the Type M 3 UO 6. Acta Crystallogr. 1966, 20, 508–513. 10.1107/s0365110x66001154.

[ref91] Hunault M. O. J. Y.; Lelong G.; Cormier L.; Galoisy L.; Solari P.-L.; Calas G. Speciation Change of Uranyl in Lithium Borate Glasses. Inorg. Chem. 2019, 58, 6858–6865. 10.1021/acs.inorgchem.9b00305. 31025856

[ref92] Amayri S.; Reich T.; Arnold T.; Geipel G.; Bernhard G. Spectroscopic Characterization of Alkaline Earth Uranyl Carbonates. J. Solid State Chem. 2005, 178, 567–577. 10.1016/j.jssc.2004.07.050.

[ref93] Arai Y.; McBeath M.; Bargar J. R.; Joye J.; Davis J. A. Uranyl Adsorption and Surface Speciation at the Imogolite-Water Interface: Self-Consistent Spectroscopic and Surface Complexation Models. Geochim. Cosmochim. Acta 2006, 70, 2492–2509. 10.1016/j.gca.2006.02.013.

[ref94] Beccia M. R.; Matara-Aho M.; Reeves B.; Roques J.; Solari P. L.; Monfort M.; Moulin C.; Den Auwer C. New Insight into the Ternary Complexes of Uranyl Carbonate in Seawater. J. Environ. Radioact. 2017, 178–179, 343–348. 10.1016/j.jenvrad.2017.08.008. 28947086

[ref95] Stewart B. D.; Mayes M. A.; Fendorf S. Impact of Uranyl - Calcium - Carbonato Complexes on Uranium(VI) Adsorption to Synthetic and Natural Sediments. Environ. Sci. Technol. 2010, 44, 928–934. 10.1021/es902194x. 20058915

[ref96] Zheng Z.; Tokunaga T. K.; Wan J. Influence of Calcium Carbonate on U(VI) Sorption to Soils. Environ. Sci. Technol. 2003, 37, 5603–5608. 10.1021/es0304897. 14717170

[ref97] Curtis G. P.; Fox P.; Kohler M.; Davis J. A. Comparison of in Situ Uranium KD Values with a Laboratory Determined Surface Complexation Model. Appl. Geochem. 2004, 19, 1643–1653. 10.1016/j.apgeochem.2004.03.004.

[ref98] Meleshyn A.; Azeroual M.; Reeck T.; Houben G.; Riebe B.; Bunnenberg C. Influence of (Calcium-)Uranyl-Carbonate Complexation on U(VI) Sorption on Ca- and Na-Bentonites. Environ. Sci. Technol. 2009, 43, 4896–4901. 10.1021/es900123s. 19673282

[ref99] Nakao A.; Ogasawara S.; Sano O.; Ito T.; Yanai J. Radiocesium Sorption in Relation to Clay Mineralogy of Paddy Soils in Fukushima, Japan. Sci. Total Environ. 2014, 468–469, 523–529. 10.1016/j.scitotenv.2013.08.062. 24055668

[ref100] Dai M.; Buesseler K. O.; Pike S. M. Plutonium in Groundwater at the 100K-Area of the U.S. DOE Hanford Site. J. Contam. Hydrol. 2005, 76, 167–189. 10.1016/j.jconhyd.2004.08.004. 15683879

[ref101] Zhuang J.; Jin Y.; Flury M. Comparison of Hanford Colloids and Kaolinite Transport in Porous Media. Vadose Zone J. 2004, 3, 395–402. 10.2113/3.2.395.

[ref102] Yang H.; Liu X.; Hao M.; Xie Y.; Wang X.; Tian H.; Waterhouse G. I. N.; Kruger P. E.; Telfer S. G.; Ma S. Functionalized Iron–Nitrogen–Carbon Electrocatalyst Provides a Reversible Electron Transfer Platform for Efficient Uranium Extraction from Seawater. Adv. Mater. 2021, 33, 2106621 10.1002/adma.202106621. 34599784

[ref103] Zhang T.; Chen J.; Xiong H.; Yuan Z.; Zhu Y.; Hu B. Constructing New Fe3O4@MnOx with 3D Hollow Structure for Efficient Recovery of Uranium from Simulated Seawater. Chemosphere 2021, 283, 131241 10.1016/j.chemosphere.2021.131241. 34470731

[ref104] Machner A.; Zajac M.; Ben Haha M.; Kjellsen K. O.; Geiker M. R.; De Weerdt K. Stability of the Hydrate Phase Assemblage in Portland Composite Cements Containing Dolomite and Metakaolin after Leaching, Carbonation, and Chloride Exposure. Cem. Concr. Compos. 2018, 89, 89–106. 10.1016/j.cemconcomp.2018.02.013.

[ref105] Dávila G.; Cama J.; Chaparro M. C.; Lothenbach B.; Schmitt D. R.; Soler J. M. Interaction between CO2-Rich Acidic Water, Hydrated Portland Cement and Sedimentary Rocks: Column Experiments and Reactive Transport Modeling. Chem. Geol. 2021, 572, 120122 10.1016/j.chemgeo.2021.120122.

