# Supplementary material for: Hydrotalcite Colloidal Stability and Interactions with Uranium(VI) at Neutral to Alkaline pH
Source: Langmuir. 2022 Feb 15;38(8):2576–89. doi: 10.1021/acs.langmuir.1c03179 (PMC9098172; doi:10.1021/acs.langmuir.1c03179)
Supplement: Supplementary file 1 — la1c03179_si_001.pdf [file la1c03179_si_001.pdf]

Supplementary Information: Hydrotalcite colloid stability and interactions with uranium(VI) at neutral to alkaline pH.

*Chris Foster[a], Samuel Shaw[a], Thomas S. Neill[a], Nick Bryan[a,b], Nick Sherriff[b], Louise S. Natrajan[c], Hannah Wilson[c], Laura Lopez-Odriozola[c], Bruce Rigby[d], Sarah J. Haigh[e], Yi-Chao Zou[e‡], Robert Harrison[f], Katherine Morris[a]**

[a] Research Centre for Radwaste Disposal and Williamson Research Centre, Department of Earth & Environmental Sciences, The University of Manchester, Oxford Road, Manchester M13 9PL, UK

*Email: katherine.morris@manchester.ac.uk. Tel.: +44 (0) 161 275 7541.

Table of Contents

Supplementary X-ray diffraction data **2**

Supplementary FTIR data **7**

Supplementary settling, ultrafiltration and zeta-potential data **10**

Supplementary X-ray Absorption Spectroscopy data **20**

Supplementary luminescence data **27**

Supplementary PHREEQC modelling **28**

Supplementary TEM images **31**

Supplementary X-ray diffraction data

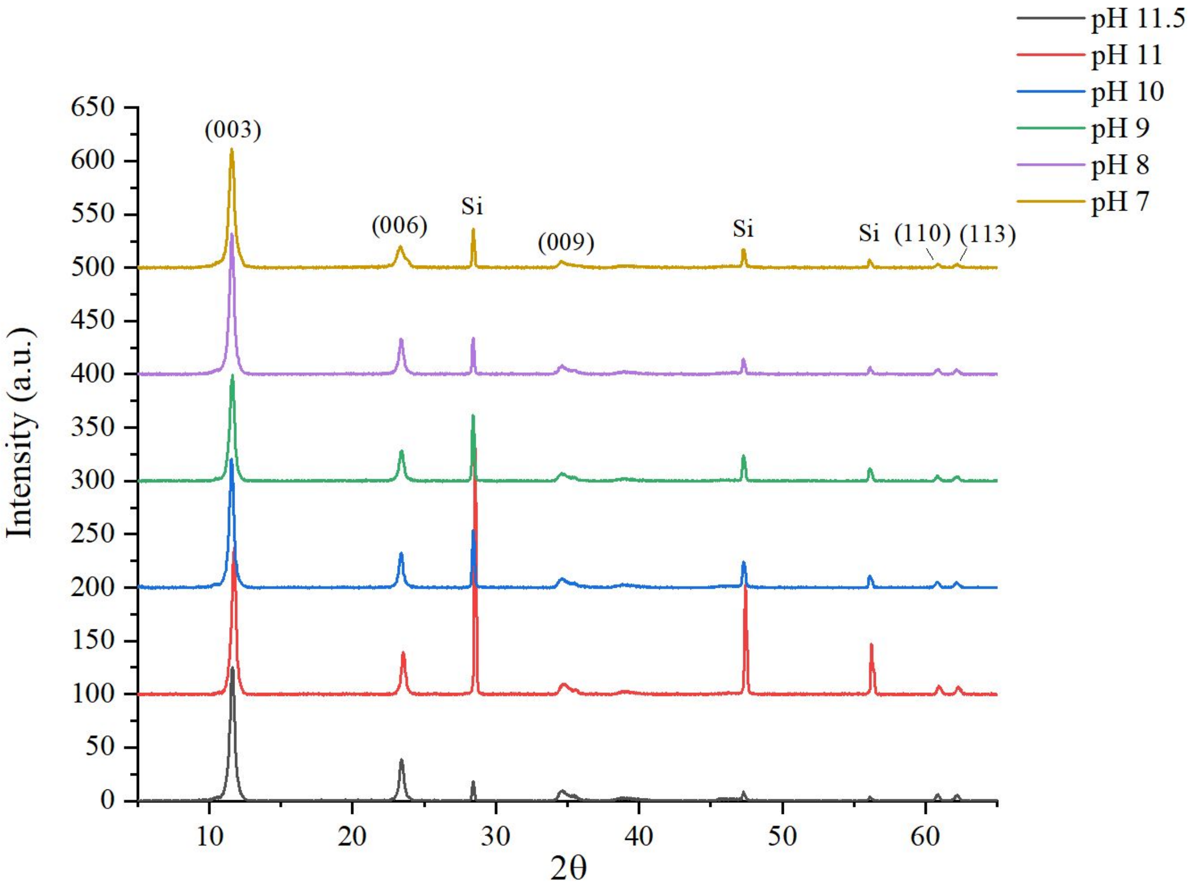

Figure S1. XRD patterns of colloidal hydrotalcite samples in the absence of U(VI) collected 1 week after the final pH adjustment. The indices of the hydrotalcite diffraction peaks hydrotalcite are given.

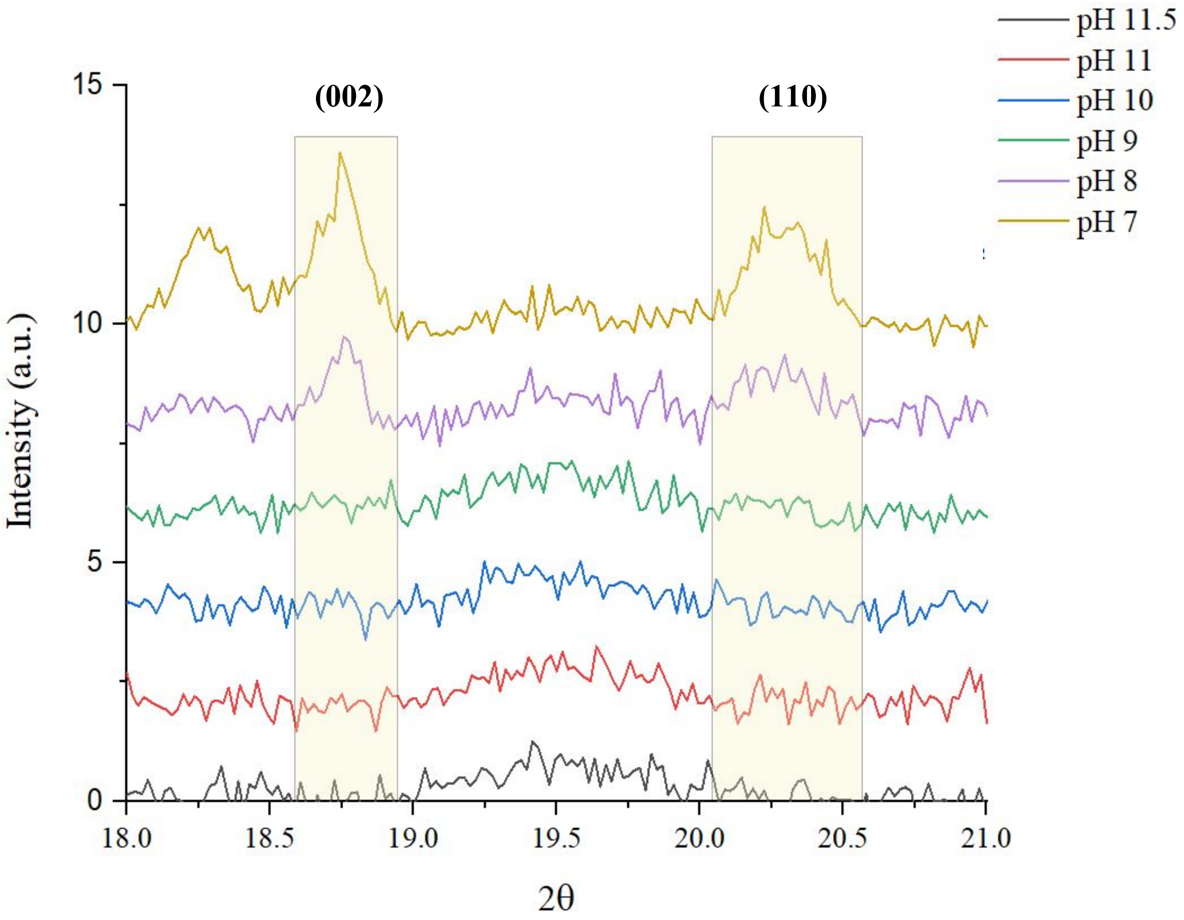

Figure S2. XRD patterns of colloidal hydrotalcite samples in the absence of U(VI) collected 1 week after the final pH adjustment. The highlighted low magnitude peaks are attributed to the presence of the Al-rich phase gibbsite (AlOOH).

Table S1. Layer-interlayer width (c) and cation-cation (a) distances for hydrotalcite samples collected from colloidal systems with varying pH and loading. Distances are calculated from the corresponding angles of the (003) and (110) peaks.

		1 hour		1 week		2 weeks		1 month	
		c (nm)	a (nm)	c (nm)	a (nm)	c (nm)	a (nm)	c (nm)	a (nm)
0 wt% U	11.5	0.761	0.304	0.762	0.304	0.761	0.304		
	11	0.754	0.304	0.756	0.304	0.762	0.305	0.760	0.304
	10	0.764	0.304	0.763	0.304	0.759	0.305	0.762	0.305
	9	0.762	0.304	0.760	0.304	0.761	0.305	0.762	0.305
	8	0.765	0.304	0.763	0.304	0.766	0.305	0.766	0.305
	7	0.764	0.304	0.764	0.304	0.765	0.304	0.766	0.305
0.01 wt% U	11.5	0.762	0.304	0.761	0.305	0.764	0.305		
	11	0.760	0.304	0.761	0.304	0.762	0.305	0.762	0.305
	10	0.764	0.304	0.763	0.304	0.762	0.305	0.764	0.305
	9	0.762	0.304	0.761	0.304	0.767	0.305	0.763	0.305
	8	0.763	0.304	0.763	0.305	0.763	0.305	0.763	0.305
	7	0.763	0.305	0.764	0.305	0.763	0.305	0.760	0.305
0.1 wt% U	11.5	0.763	0.305	0.763	0.305	0.762	0.305		
	11	0.765	0.305	0.763	0.305	0.761	0.304	0.764	0.304
	10	0.760	0.305	0.760	0.304	0.762	0.305	0.762	0.305
	9	0.762	0.304	0.762	0.305	0.766	0.305	0.764	0.304
	8	0.762	0.304	0.764	0.304	0.764	0.305	0.765	0.304
	7	0.759	0.305	0.765	0.305	0.765	0.305	0.765	0.305
1 wt% U	11.5	0.765	0.305	0.765	0.305	0.765	0.304		
	11	0.767	0.305	0.770	0.305	0.766	0.304	0.770	0.304
	10	0.765	0.304	0.768	0.305	0.766	0.305	0.766	0.305
	9	0.764	0.305	0.771	0.305	0.766	0.304	0.768	0.305
	8	0.769	0.305	0.767	0.304	0.772	0.305	0.768	0.305
	7	0.761	0.305	0.771	0.304	0.770	0.305	0.768	0.305

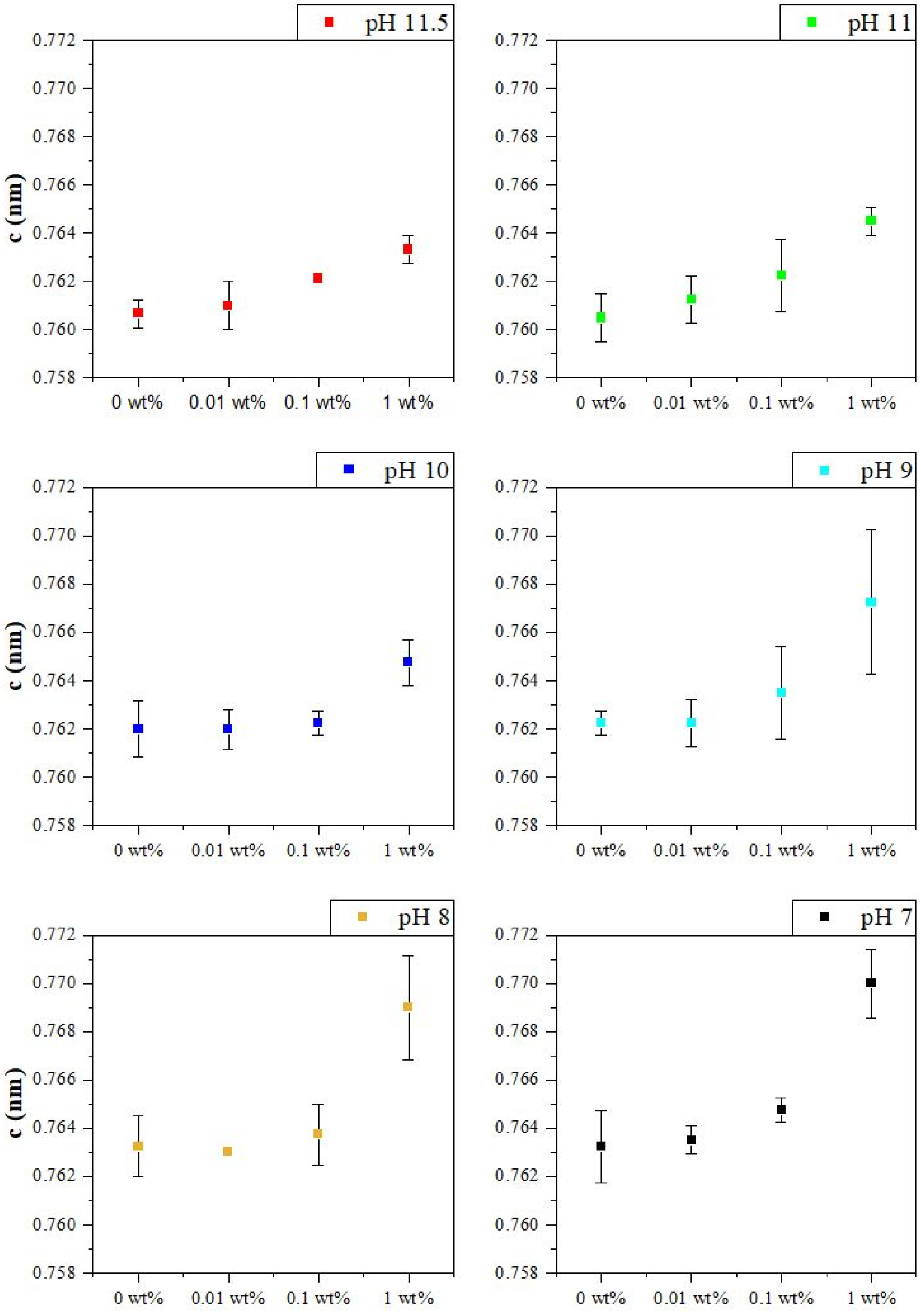

Figure S3. Changes in the layer-interlayer width (c) with variable U(VI) surface loading at differing pHs (7 – 11.5). Error bars represent standard deviation of the layer-interlayer width which was averaged using the distances recorded at each timepoint (1h, 1w, 2w and 1m) for the different pH colloidal systems.

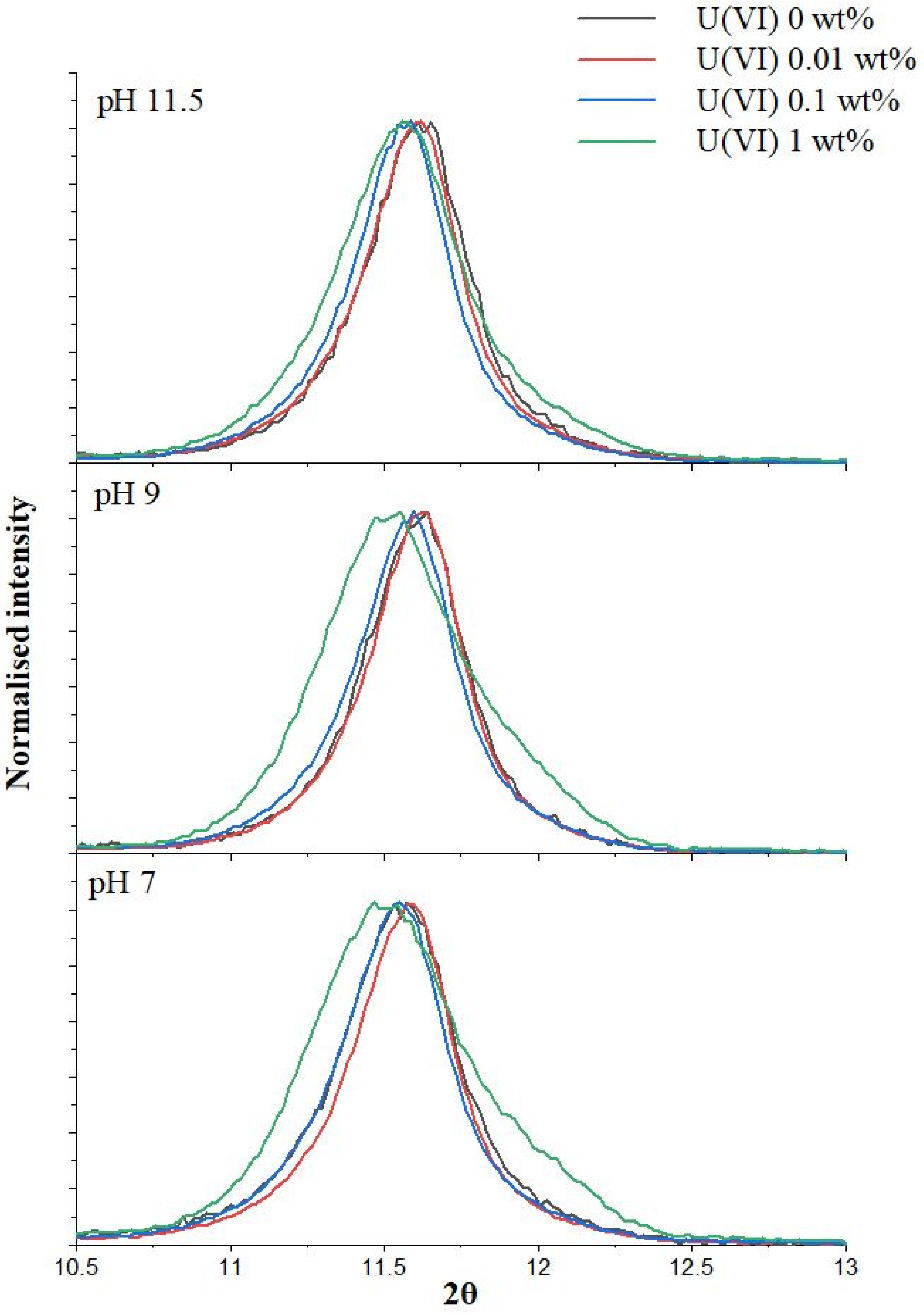

Figure S4. XRD spectra of hydrotalcite samples collected from colloidal suspensions at pH 11.5, 9 and 7 1 week after the final pH adjustment. The spectra have been normalised with respect to the (003) peak to compare the extents of broadening.

Supplementary FTIR data

Table S2. Key, recurring IR peaks and their corresponding bond vibrations identified in the colloidal hydrotalcite samples collected from systems with variable pH and U(VI) loading.

**Wavenumber range/ cm^{-1}**	**Bond vibration**	
3360 - 3370	O-H	Stretches and bending of surface hydroxyl groups
3260 - 3270	O-H	Stretches of hydrogen-bonded H_2O in the interlayer
1650 - 1660	O-H	Free H_2O molecules in the interlayer
1360 - 1380	C-O	Interlayer carbonate anions
<1000	Mg-O, Al-O	Bond vibrations within the brucite-like layers

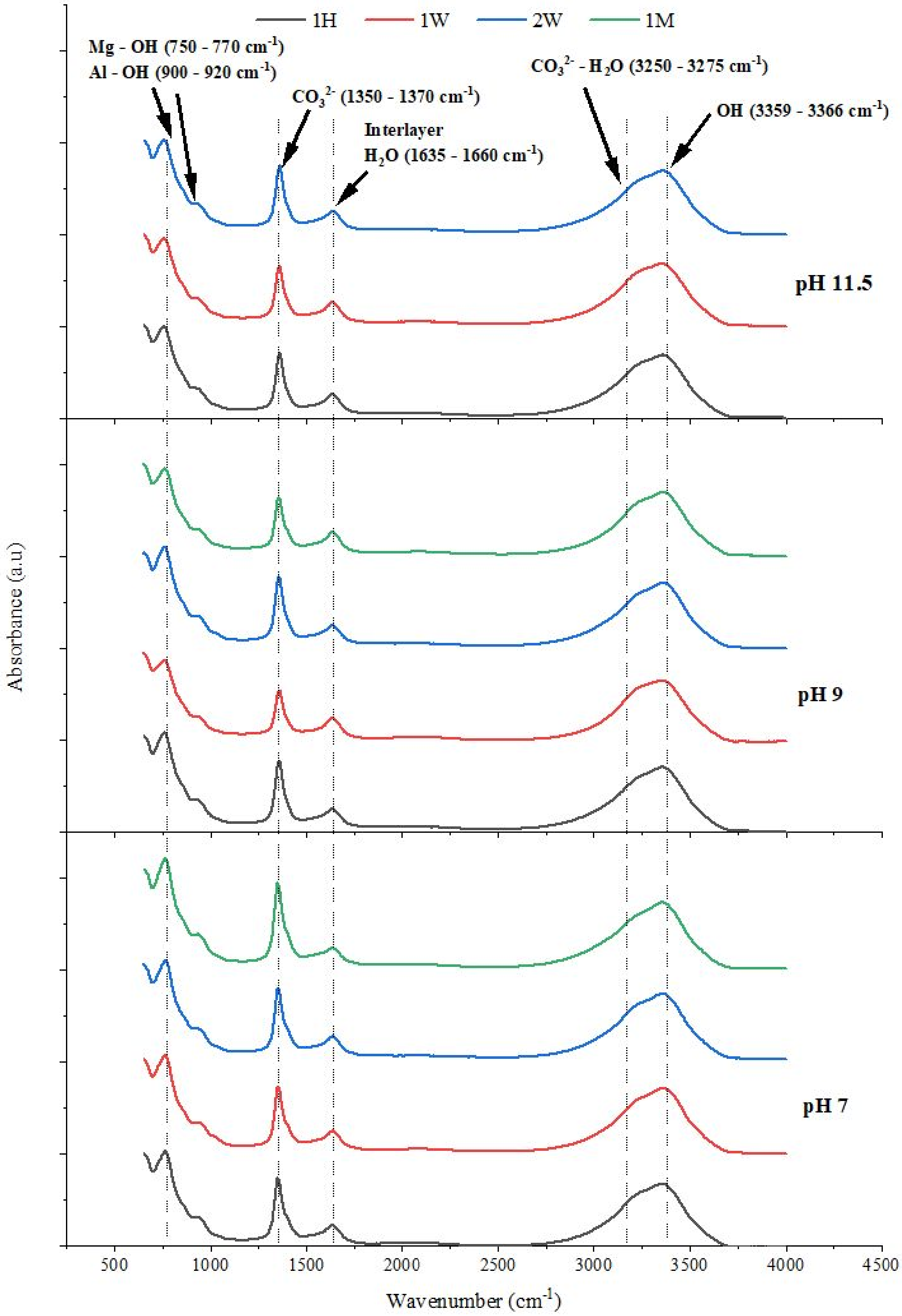

Figure S5. FTIR spectra of colloidal hydrotalcite samples collected from different pH systems and at different timepoints.

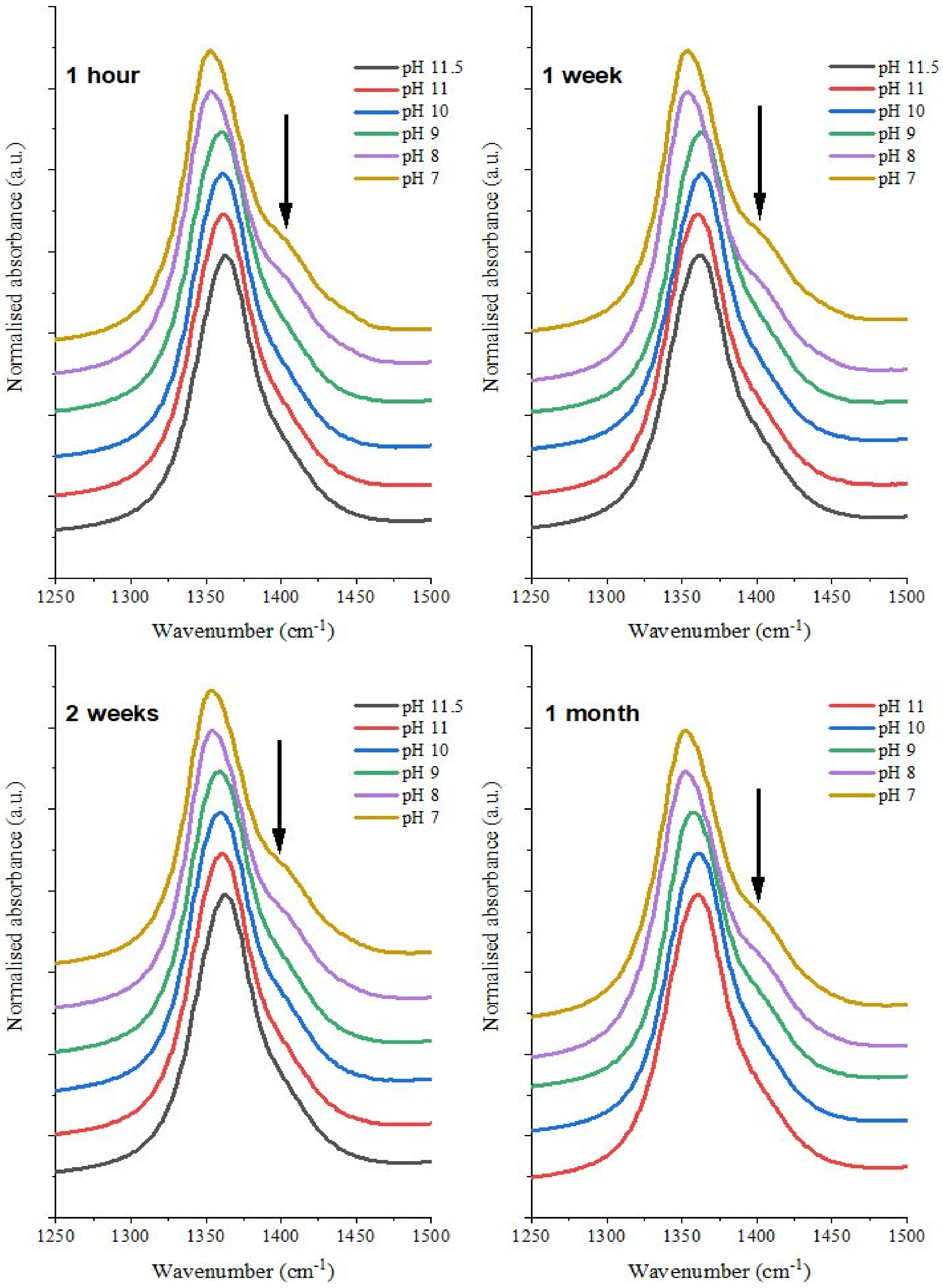

Figure S6. Comparison of the normalised carbonate stretching peak of samples taken from colloidal hydrotalcite systems of varying pH. The arrow points out the emergence of a shoulder at ~1400 cm^{-1} for samples removed from the pH 8 and 7 systems, a result of changes in the symmetry of the interlayer carbonate.

Supplementary settling, ultrafiltration and zeta-potential data

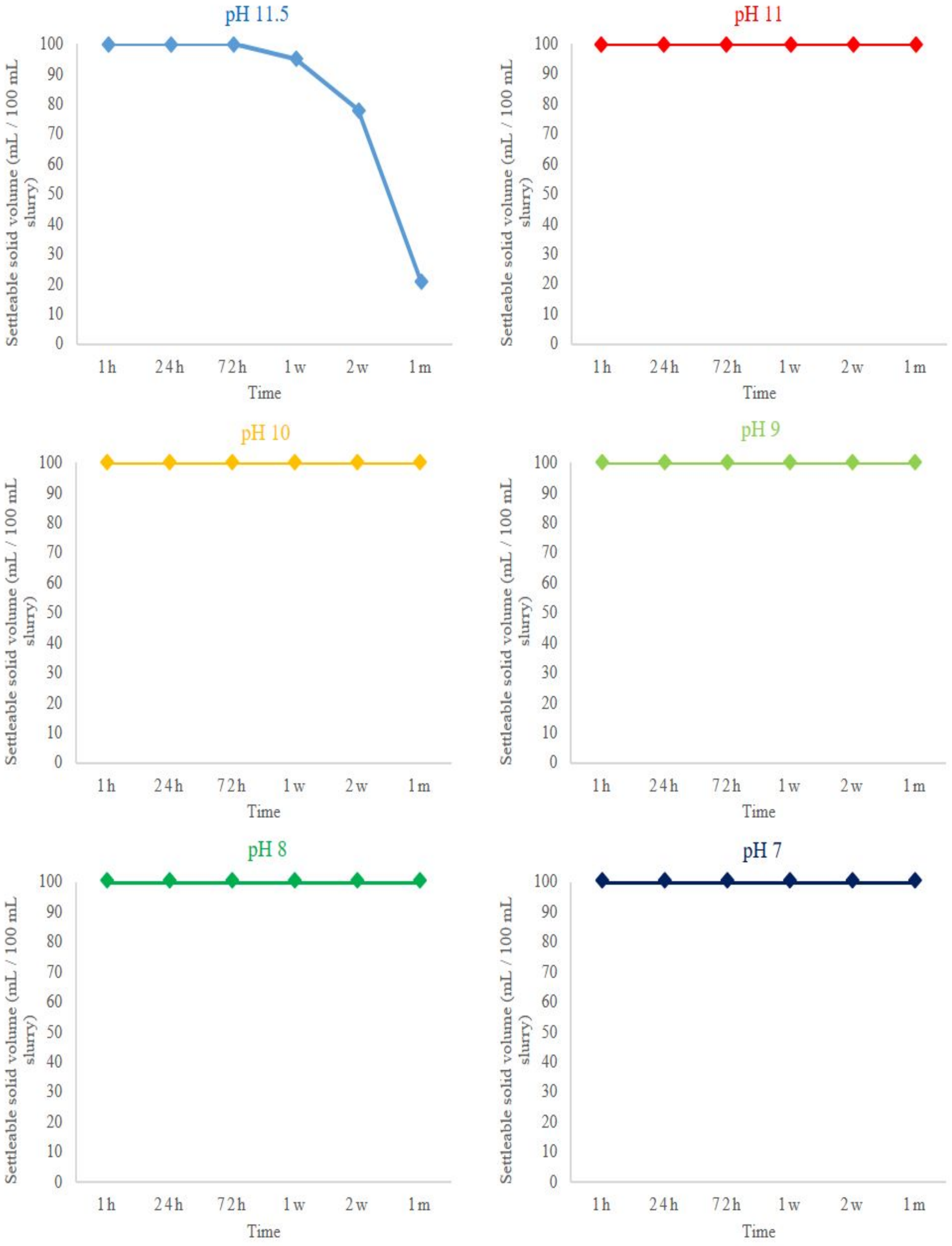

Figure S7. Hydrotalcite settleable solid volume tracked over the course a month. Colloidal systems were adjusted so that the initial pHs are 11.5, 11, 10, 9, 8 and 7.

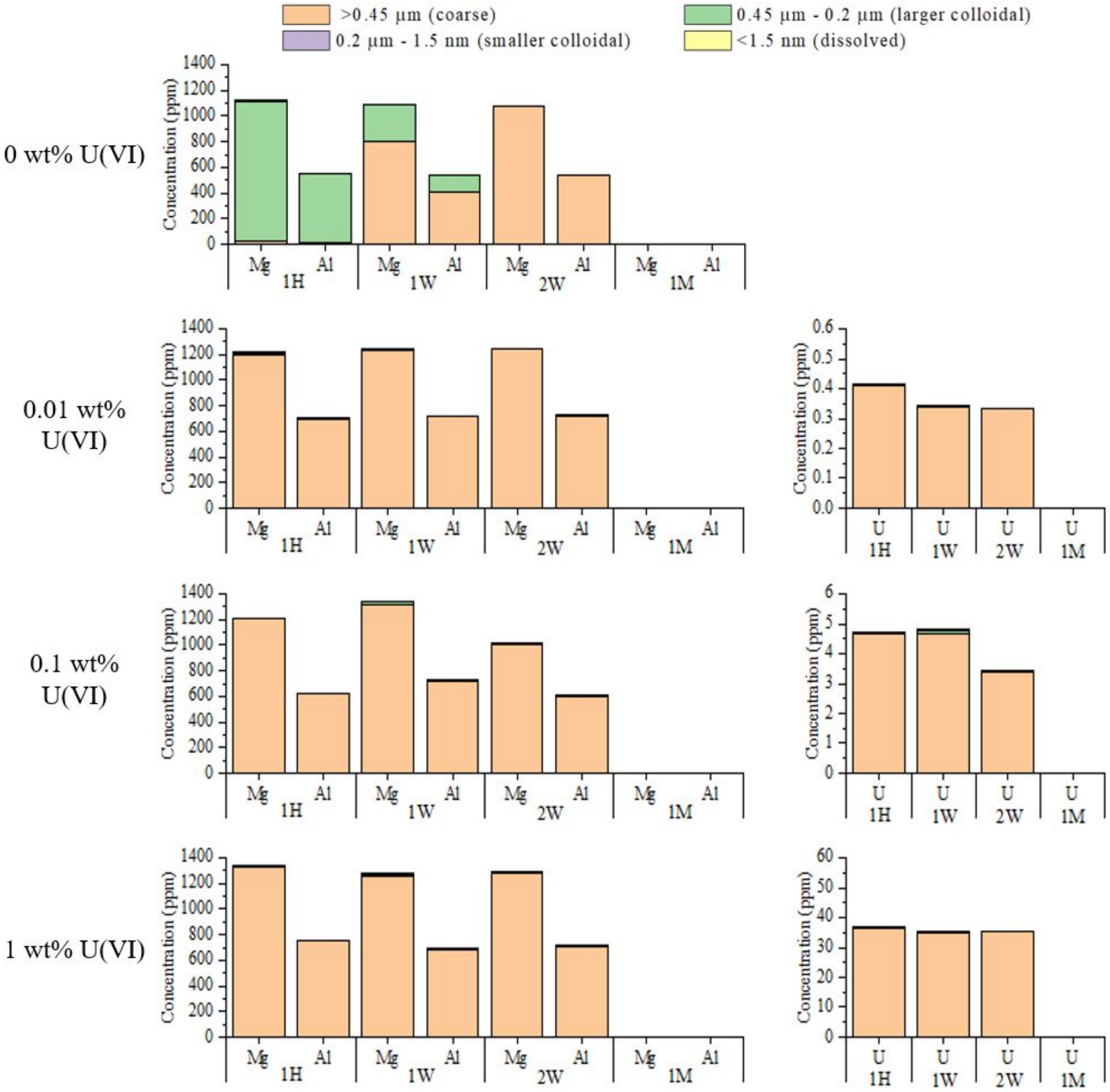

Figure S8. Ultrafiltration data highlighting the changes in hydrotalcite colloidal particle size distribution in the supernatant at pH 11.5 and with varying U(VI) surface loading over the course of a month. The different size ranges are referred to as the coarse (>0.45 μm), larger colloidal (0.2 μm - 0.45 μm), smaller colloidal (1.5 nm - 0.2 μm) and dissolved (<1.5 nm) fractions.

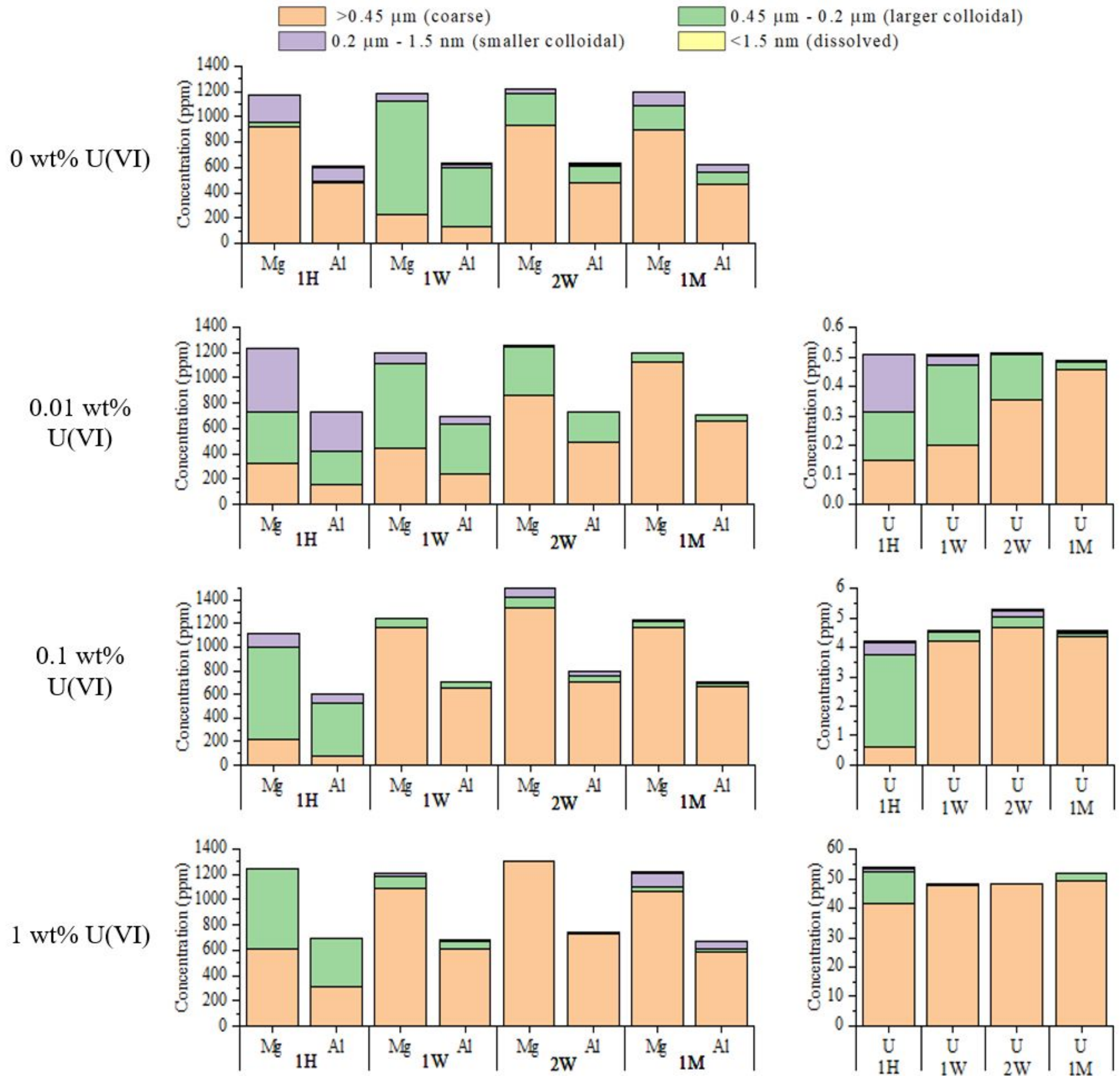

Figure S9. Ultrafiltration data highlighting the changes in hydrotalcite colloidal particle size distribution in the supernatant at pH 11 and with varying U(VI) surface loading over the course of a month. The different size ranges are referred to as the coarse (>0.45 µm), larger colloidal (0.2 µm - 0.45 µm), smaller colloidal (1.5 nm - 0.2 µm) and dissolved (<1.5 nm) fractions.

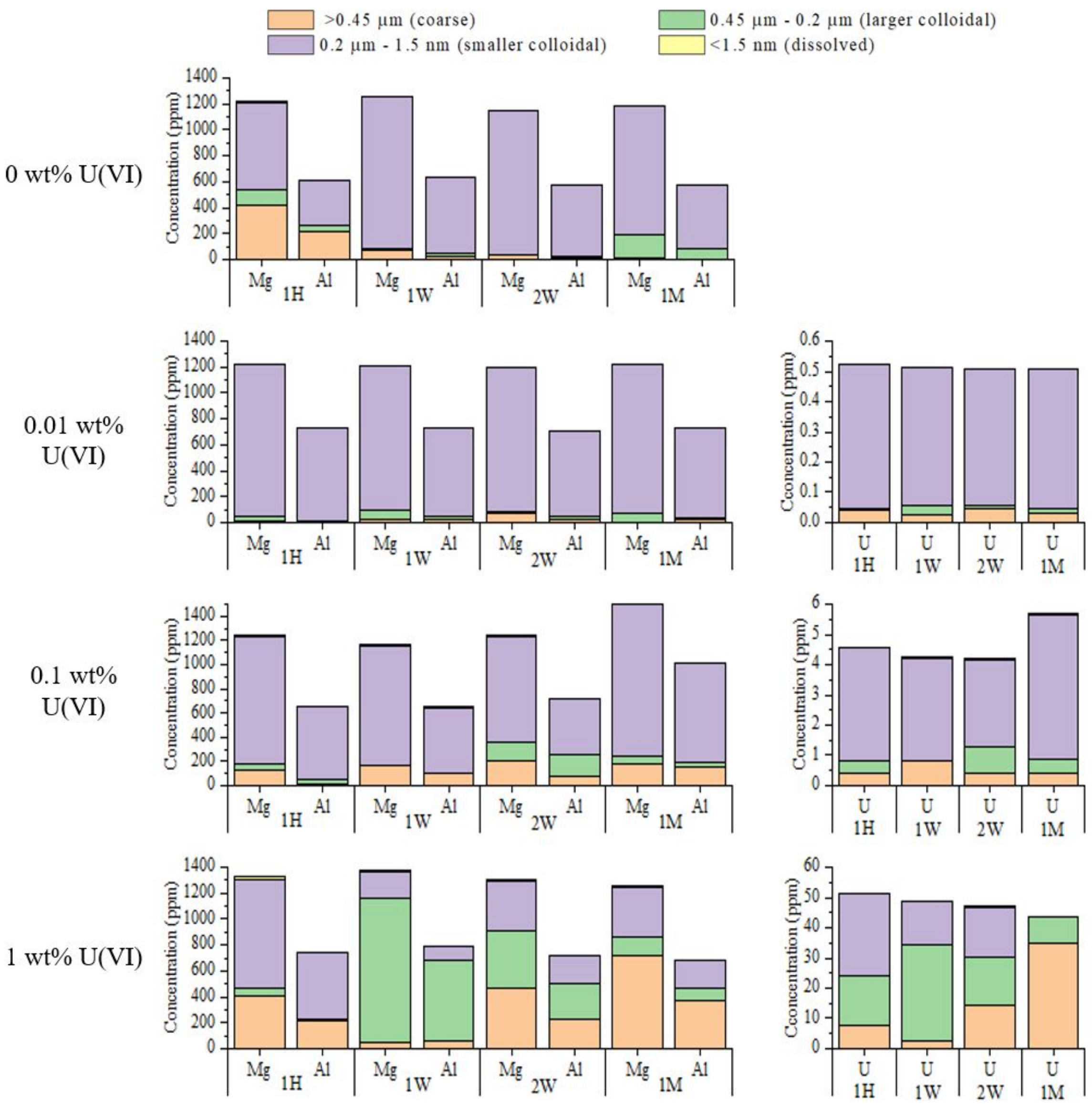

Figure S10. Ultrafiltration data highlighting the changes in hydrotalcite colloidal particle size distribution in the supernatant at pH 10 and with varying U(VI) surface loading over the course of a month. The different size ranges are referred to as the coarse (>0.45 µm), larger colloidal (0.2 µm - 0.45 µm), smaller colloidal (1.5 nm - 0.2 µm) and dissolved (<1.5 nm) fractions.

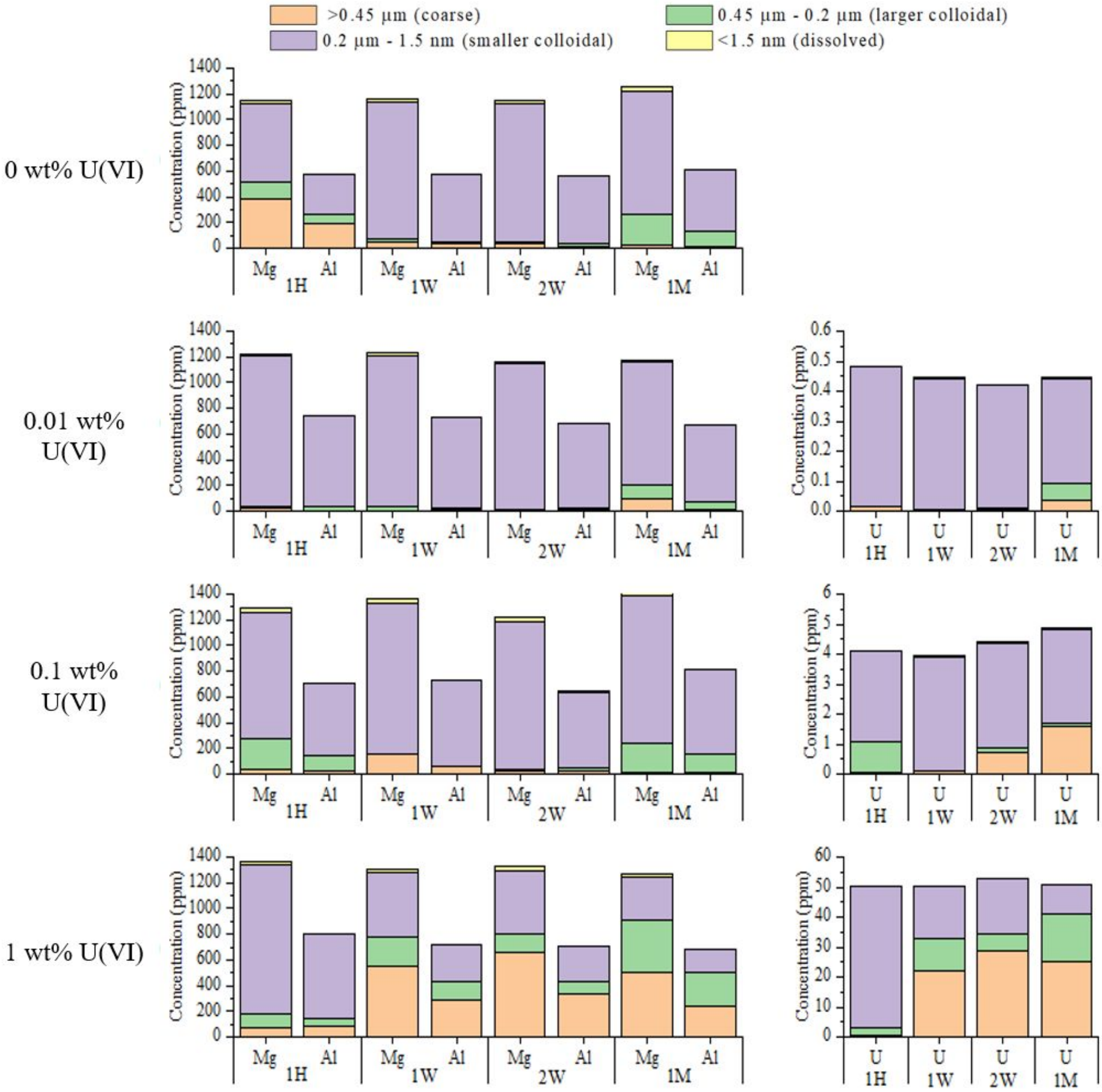

Figure S11. Ultrafiltration data highlighting the changes in hydrotalcite colloidal particle size distribution in the supernatant at pH 9 and with varying U(VI) surface loading over the course of a month. The different size ranges are referred to as the coarse (>0.45 µm), larger colloidal (0.2 µm - 0.45 µm), smaller colloidal (1.5 nm - 0.2 µm) and dissolved (<1.5 nm) fractions.

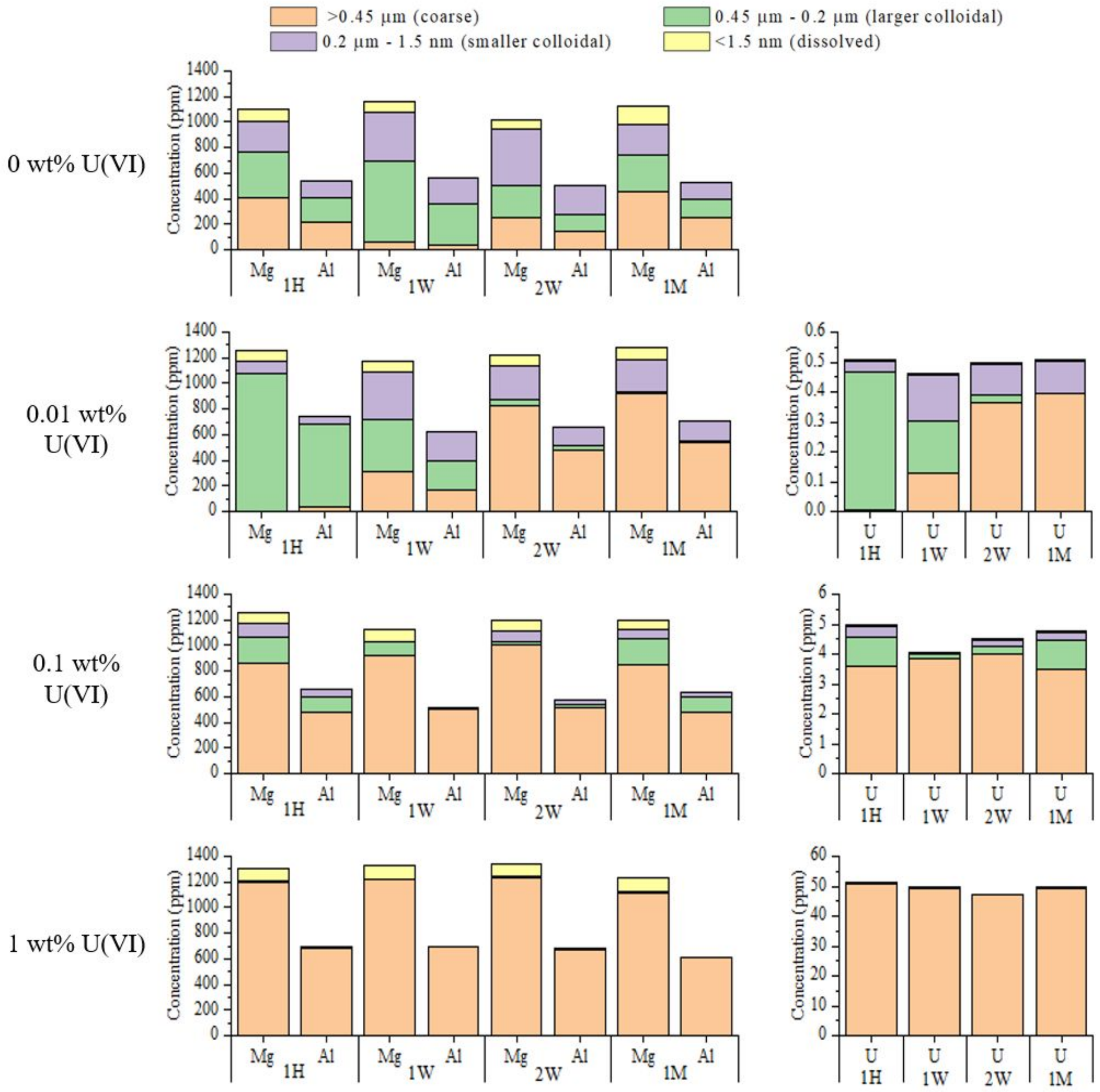

Figure S12. Ultrafiltration data highlighting the changes in hydrotalcite colloidal particle size distribution in the supernatant at pH 8 and with varying U(VI) surface loading over the course of a month. The different size ranges are referred to as the coarse (>0.45 µm), larger colloidal (0.2 µm - 0.45 µm), smaller colloidal (1.5 nm - 0.2 µm) and dissolved (<1.5 nm) fractions.

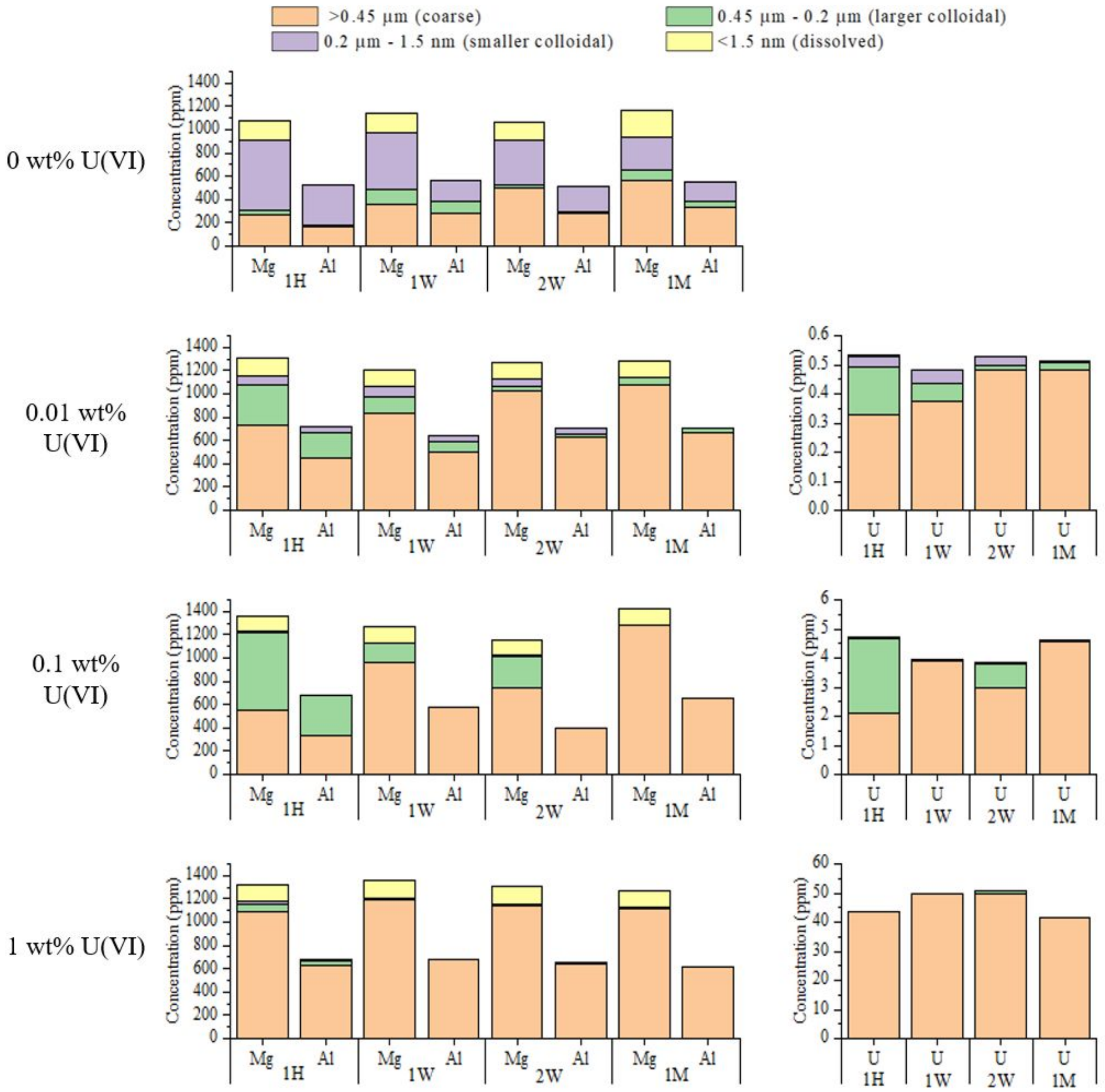

Figure S13. Ultrafiltration data highlighting the changes in hydrotalcite colloidal particle size distribution in the supernatant at pH 7 and with varying U(VI) surface loading over the course of a month. The different size ranges are referred to as the coarse (>0.45 µm), larger colloidal (0.2 µm - 0.45 µm), smaller colloidal (1.5 nm - 0.2 µm) and dissolved (<1.5 nm) fractions.

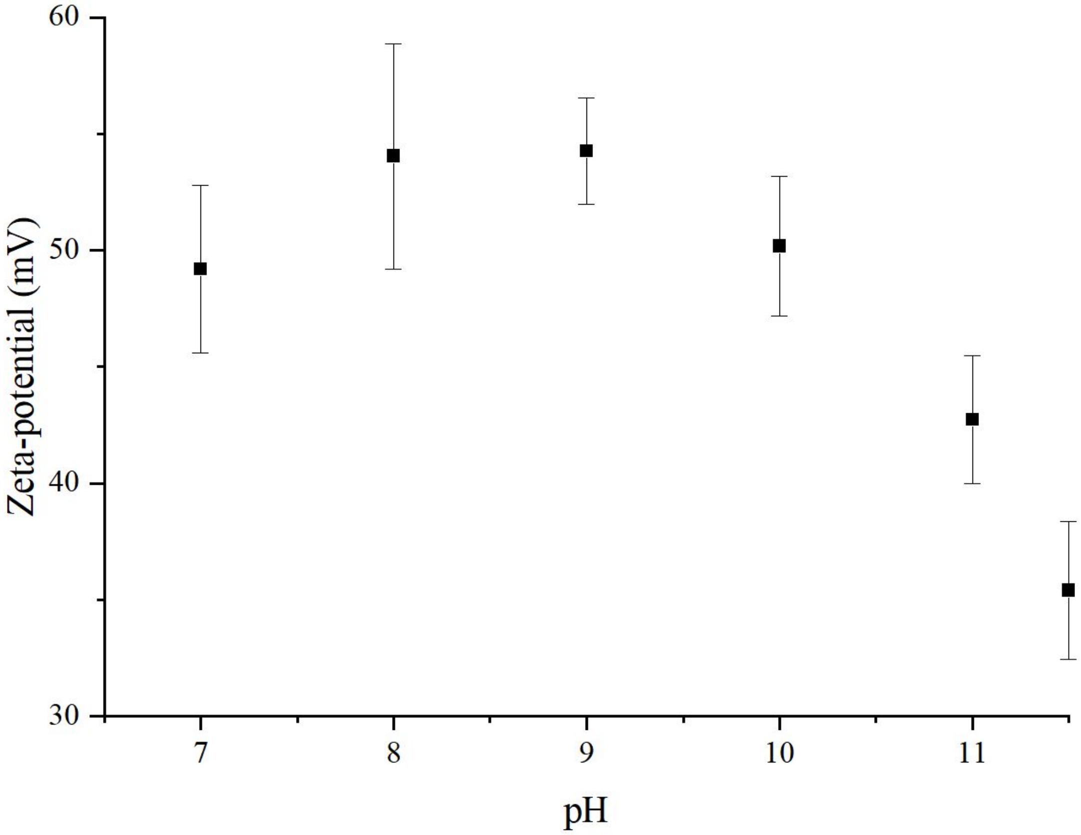

Figure S14. The average zeta-potential measurements for the hydrotalcite colloid systems at varying pH over the course of a month. Errors calculated from the standard deviation of the measurements taken at each timepoint.

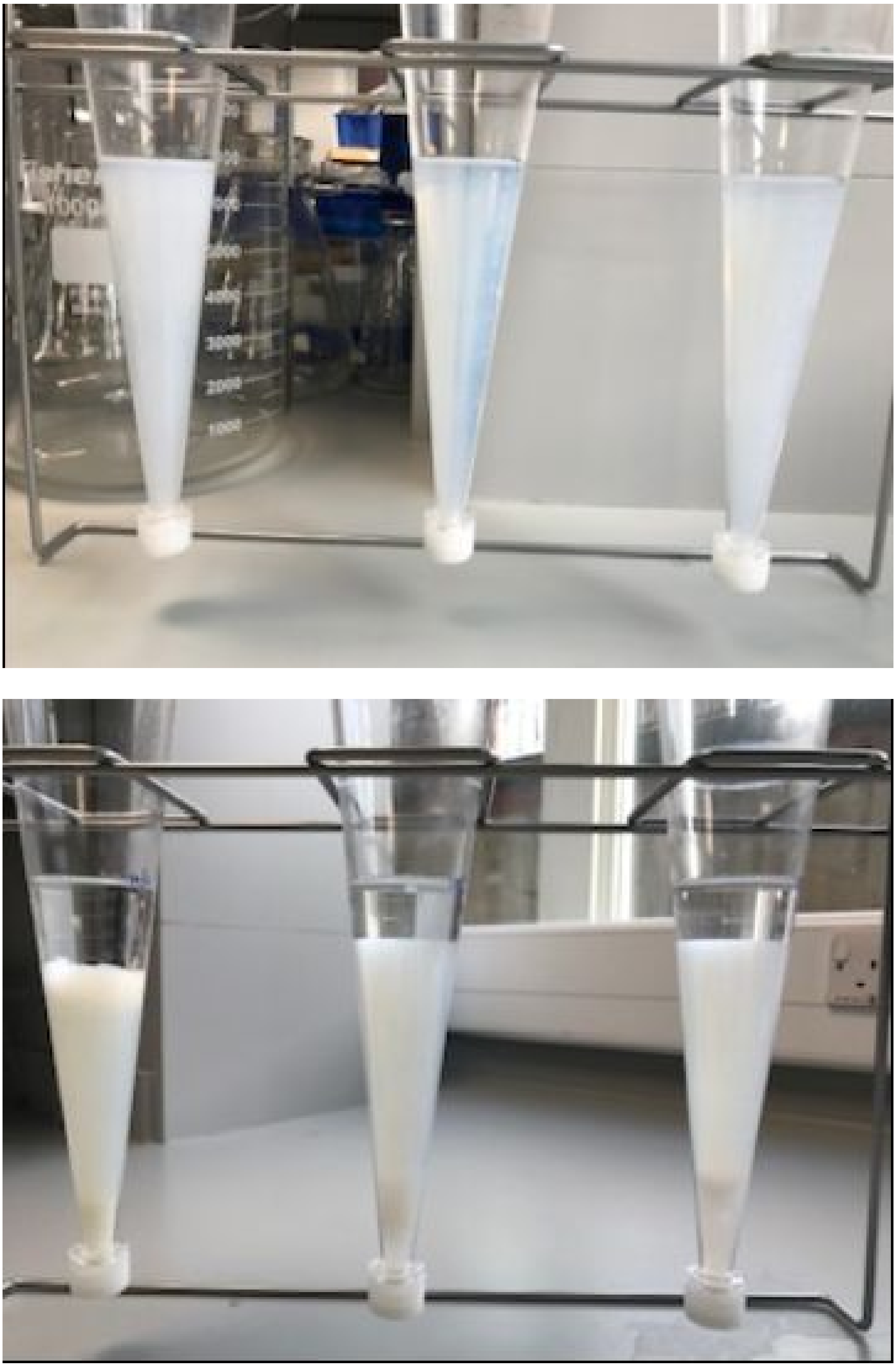

Figure S15. Top. 1 hour timepoint for the settling experiments involving the pH 11.5 1 w% (left), 0.1 wt% (middle) and 0.01 wt% (right) colloidal systems. **Bottom.** 2 week timepoint for the settling experiments.

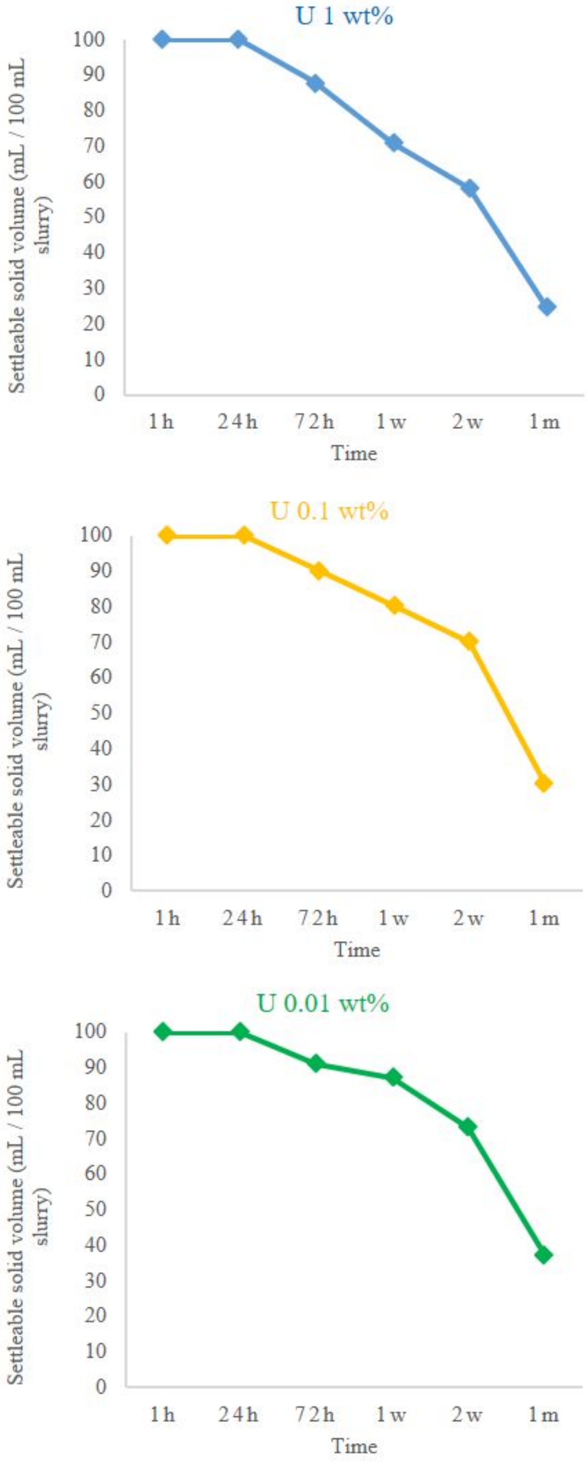

.

Figure S16. Hydrotalcite settleable solid volume tracked over the course a month at pH 11.5. Colloidal systems were adjusted to contain three different U(VI) loadings,0.01, 0.1 and 1.0 wt % U with respect to initial colloid loading.

Supplementary X-ray Absorption Spectroscopy data

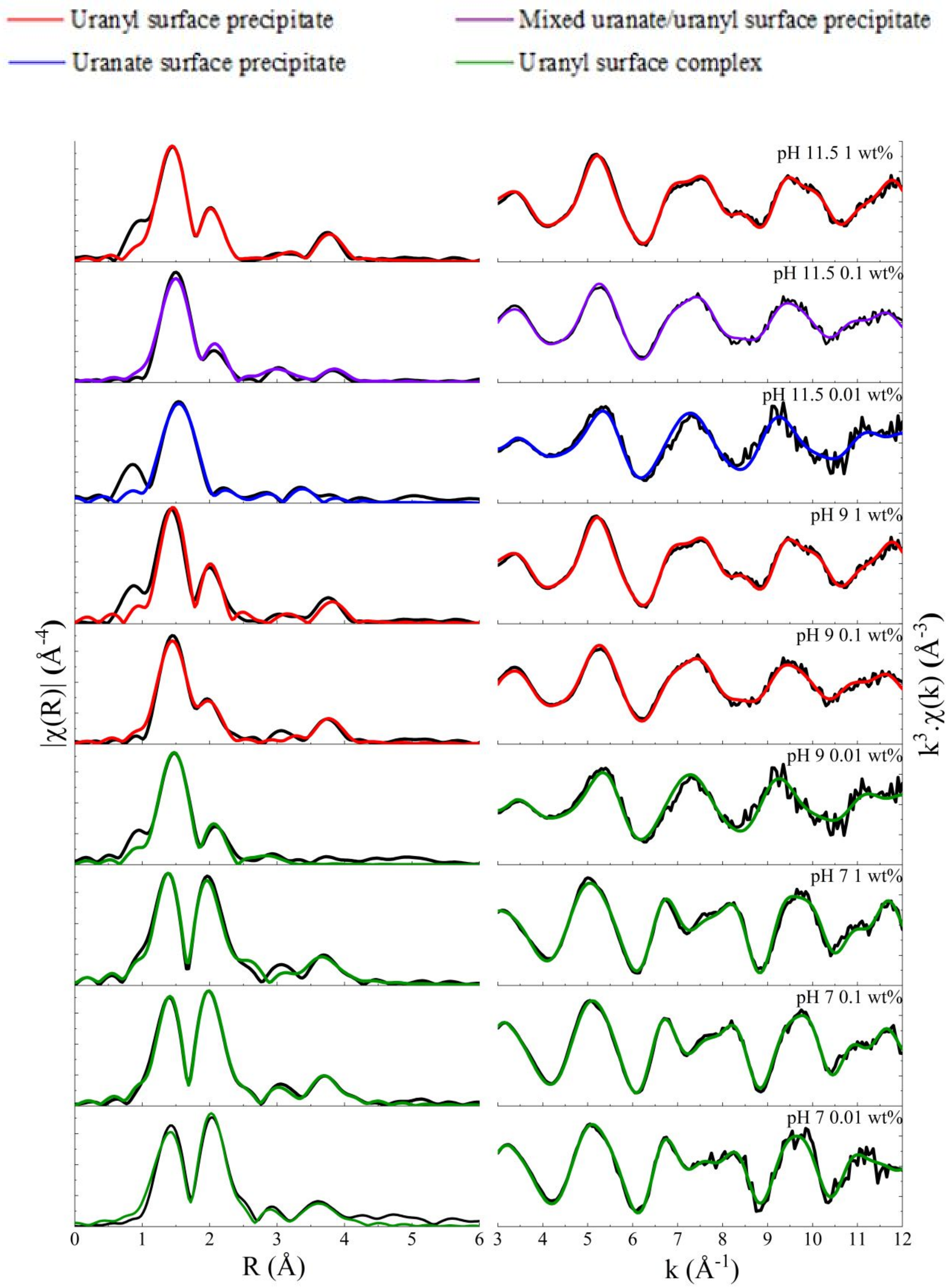

Figure S17. U L_3-edge EXAFS spectra for U(VI) adsorbed onto hydrotalcite for 24 hours and at variable pH and U loading. **Right.** k^3-weighted EXAFS (black lines are raw data, coloured lines are corresponding fits). **Left.** Fourier transform of k^3-weighted EXAFS.

Table S3. The fitting parameters for the 1 wt% samples at pH 11.5, 9 and 7, including coordination numbers (N), U interatomic distances (R (Å)), Debye-Waller factors (σ^2), shift

in energy from calculated Fermi level (ΔE_0) and 'goodness of fit' factor (R). Numbers in parentheses are the standard deviation on the last decimal place. a indicates that fixed R values were used for the associated path and c indicates tied σ^2 values. Multiple scattering paths are denoted by MS and were defined using parameters previously used for single scattering paths.

**Sample**	**Path**	**ΔE_0 (eV)**	**CN**	**R(Å)**	**σ^2(Å^2)**	**$S0_2$**	**R-factor**
	U-O$_{ax}$	4.9(15)	2.0	1.80(1)	0.003(1)		
	U-Oeq$_1$^{a}	4.9(15)	2.0	2.21(2)	0.002(1)^b		
	U-Oeq$_2$	4.9(15)	2.0	2.38(2)	0.002(1)^b		
	U-Oeq$_3$	4.9(15)	1.0	2.44(2)	0.002(1)^b		
**pH 11.5 1wt%**	U-O	4.9(15)	1.0	2.86(2)	0.003(3)	1.0	0.006
	U-U	4.9(15)	3.0	3.87(2)	0.007(2)		
	U-O$_{ax}$^{MS}	4.9(15)	2.0	3.66(2)	0.012(0)		
	U-O$_{ax}$^{MS}	4.9(15)	2.0	3.61(2)	0.006(0)		
	U-O$_{ax}$^{MS}	4.9(15)	2.0	3.61(2)	0.006(0)		
	U-O$_{ax}$	10.5(16)	2.0	1.82(1)	0.003(1)		
	U-Oeq$_1$^{a}	10.5(16)	1.5	2.22(3)	0.006(2)^b		
	U-Oeq$_2$	10.5(16)	3.5	2.41(3)	0.006(2)^b		
**pH 9 1wt%**	U-O	10.5(16)	1.0	2.90(5)	0.006(6)	1.0	0.016
	U-U	10.5(16)	3.0	3.87(3)	0.008(3)		
	U-O$_{ax}$^{MS}	10.5(16)	2.0	3.56(2)	0.012(2)		
	U-O$_{ax}$^{MS}	10.5(16)	2.0	3.56(2)	0.006(2)		
	U-O$_{ax}$^{MS}	10.5(16)	2.0	3.56(2)	0.006(2)		
	U-O$_{ax}$	8.2(11)	2.0	1.81(1)	0.004(1)		
	U-Oeq$_1$	8.2(11)	5.0	2.45(1)	0.005(1)		
	U-C	8.2(11)	3.0	2.92(1)	0.002(2)		
	U-O$_{dis}$	8.2(11)	2.0	4.09(2)	0.005(4)		
**pH 7 1wt%**	U-O$_{ax}$^{MS}	8.2(11)	2.0	3.70(2)	0.015(2)	1.0	0.014
	U-O$_{ax}$^{MS}	8.2(11)	2.0	3.63(2)	0.008(2)		
	U-O$_{ax}$^{MS}	8.2(11)	2.0	3.63(2)	0.008(2)		
	U-O$_{ax}$-O$_{eq}$-U^{MS}	8.2(11)	16.0	3.66(2)	0.004(2)		
	U-C-O$_{dis}$-U^{MS}	8.2(11)	6.0	4.14(2)	0.004(2)		
	U-C-O$_{dis}$-C-U^{MS}	8.2(11)	3.0	4.15(2)	0.004(2)		

Table S4. The fitting parameters for the 0.1 wt% samples at pH 11.5, 9 and 7, including coordination numbers (N), U interatomic distances (R (Å)), Debye-Waller factors (σ^2), shift in energy from calculated Fermi level (ΔE_0) and 'goodness of fit' factor (R). Numbers in parentheses are the standard deviation on the last decimal place. a indicates that fixed R values were used for the associated path, b indicates a constrained parameter and c indicates tied σ^2 values. Multiple scattering paths are denoted by MS and were defined using parameters previously used for single scattering paths.

Sample	Path	E_0	CN	R(Å)	σ^2(Å^2)	$S0_2$	R-factor
**pH 11.5 0.1wt%**	U-O_{ax}	9.9(18)	2.0	1.83(1)	0.004(1)	1.0	0.016
	U-Oeq_1	9.9(18)	2.5	2.26(2)	0.003(2)^b		
	U-Oeq_2	9.9(18)	3.0	2.44(2)	0.003(2)^b		
	U-Na	9.9(18)	1.3	3.42(5)	0.006(5)		
	U-U	9.9(18)	1.8	3.92(4)	0.008(4)		
	U-O_{ax}^{MS}	9.9(18)	2.0	3.66(2)	0.016(2)		
	U-O_{ax}^{MS}	9.9(18)	2.0	3.63(2)	0.008(2)		
	U-O_{ax}^{MS}	9.9(18)	2.0	3.63(2)	0.008(2)		
**pH 9 0.1wt%**	U-O_{ax}	8.7(19)	2.0	1.82(1)	0.004(1)	1.0	0.018
	U-Oeq_1	8.7(19)	1.5	2.24(3)	0.003(3)^b		
	U-Oeq_2	8.7(19)	2.0	2.36(2)	0.003(3)^b		
	U-Oeq_2	8.7(19)	2.0	2.47(2)	0.003(3)^b		
	U-U	8.7(19)	3.0	3.87(3)	0.008(2)		
	U-O_{ax}^{MS}	8.7(19)	2.0	3.61(2)	0.016(2)		
	U-O_{ax}^{MS}	8.7(19)	2.0	3.61(2)	0.008(2)		
	U-O_{ax}^{MS}	8.7(19)	2.0	3.61(2)	0.008(2)		
**pH 7 0.1wt%**	U-O_{ax}	11.8(3)	2.0	1.82(3)	0.004(0)	1.0	0.019
	U-Oeq_1	11.8(3)	5.5	2.46(3)	0.004(2)		
	U-C	11.8(3)	2.3	2.92(1)	0.001(1)		
	U-Mg	11.8(3)	1.5	3.33(5)	0.004(1)		
	U-O_{dis}	11.8(3)	1.0	4.12(5)	0.007(2)		
	U-O_{ax}^{MS}	11.8(3)	2.0	3.72(6)	0.014(2)		
	U-O_{ax}^{MS}	11.8(3)	2.0	3.64(3)	0.007(2)		
	U-O_{ax}^{MS}	11.8(3)	2.0	3.64(3)	0.007(2)		

Table S5. The fitting parameters for the 0.01 wt% samples at pH 11.5, 9 and 7, including coordination numbers (N), U interatomic distances (R (Å)), Debye-Waller factors (σ^2), shift in energy from calculated Fermi level (ΔE_0) and 'goodness of fit' factor (R). Numbers in parentheses are the standard deviation on the last decimal place. a indicates that fixed R values were used for the associated path, b indicates a constrained parameter and c indicates tied σ^2 values. Multiple scattering paths are denoted by MS and were defined using parameters previously used for single scattering paths.

Sample	Path	E_0	CN	R(Å)	σ^2(Å^2)	$S0_2$	R-factor
**pH 11.5 0.01wt%**	U-O_{ax}	9.8(15)	2.0	1.86(1)	0.004(1)	1.0	0.010
	U-Oeq_1	9.8(15)	3.0	2.26(2)	$0.004(1)^b$		
	U-Oeq_1	9.8(15)	1.5	2.44(1)	$0.004(1)^b$		
	U-Na	9.8(15)	2.0	3.31(3)	$0.006(3)^b$		
	U-Na	9.8(15)	2.0	3.54(3)	$0.006(3)^b$		
	U-O_{ax}^{MS}	9.8(15)	2.0	3.67(2)	0.016(2)		
	U-O_{ax}^{MS}	9.8(15)	2.0	3.72(2)	0.008(2)		
	U-O_{ax}^{MS}	9.8(15)	2.0	3.72(2)	0.008(2)		
**pH 9 0.01wt%**	U-O_{ax}	7.1(30)	2.0	1.83(1)	0.003(0)	1.0	0.010
	U-Oeq_1	7.1(30)	2.5	2.24(3)	$0.005(2)^b$		
	U-Oeq_2	7.1(30)	3.0	2.43(3)	$0.005(2)^b$		
	U-Mg	7.1(30)	1.0	3.29(5)	0.007(6)		
**pH 7 0.01wt%**	U-O_{ax}	10.9(7)	2.0	1.81(0)	0.005(1)	1.0	0.017
	U-Oeq_1	10.9(7)	1.5	2.29(1)	$0.002(1)^b$		
	U-Oeq_2	10.9(7)	4.5	2.46(1)	$0.002(1)^b$		
	U-C	10.9(7)	2.2	2.88(4)	0.005(5)		
	U-Mg	10.9(7)	1.3	3.31(5)	0.008(7)		
	U-O_{dis}	10.9(7)	1.0	4.15(5)	0.005(4)		
	U-O_{ax}^{MS}	10.9(7)	2.0	3.68(1)	0.015(2)		
	U-O_{ax}^{MS}	10.9(7)	2.0	3.68(1)	0.008(2)		
	U-O_{ax}^{MS}	10.9(7)	2.0	3.68(1)	0.008(2)		

Table S6. Published EXAFS fitting parameters and x-ray diffraction crystallographic distances for U(VI) minerals.

	EXAFS fitting			XRD
	**Path**	**N**	**R(Å)**	**R(Å)**
**Compreignacite[a] ($Na_2(UO_2)_6O_4(OH)_6$:$7H_2O$)**	U-O_{ax}	2	1.82	1.8
	U-O_{eq}	2	2.28	2.24
	U-O_{eq}	2	2.46	2.43
	U-O_{eq}	2/3	2.65	2.57
	U-O_{eq}	1/3	2.85	2.86
	U-U	4/3	3.82	3.82
	U-U	4/3	3.88	3.88
	U-U	4/3	3.94	3.94
	U-U	2	4.65	4.62
**Sodium uranate[b] ($Na_2U_2O_7$)**	U-O_{ax}	2	1.87	1.89
	U-O_{eq}	4.5	2.22	2.3
	U-O_{eq}	1	2.54	
	U-Na	1	3.54	3.73
	U-U	3	3.83	3.95
	U-U	1	4.3	
**Liebigite[a] $Ca_2(UO_2)(CO_3)_3 \cdot 11H_2O$**	U-O_{ax}	2	1.8	1.77
	U-O_{eq}	6	2.42	2.43
	U-C	3	2.88	2.89
	U-Odis	3	4.15	4.13
	MS	6	4.15	4.13
	MS	3	4.15	4.13

[a] *Catalano, J. G.; Brown, G. E. Analysis of Uranyl-Bearing Phases by EXAFS Spectroscopy: Interferences, Multiple Scattering, Accuracy of Structural Parameters, and Spectral Differences. Am. Mineral.* ***2004****.* https://doi.org/10.2138/am-2004-0711.
[b] *Bots, P.; Morris, K.; Hibberd, R.; Law, G. T. W.; Mosselmans, J. F. W.; Brown, A. P.; Doutch, J.; Smith, A. J.; Shaw, S. Formation of Stable Uranium(VI) Colloidal Nanoparticles in Conditions Relevant to Radioactive Waste Disposal. Langmuir* ***2014****. https://doi.org/10.1021/la502832j.*

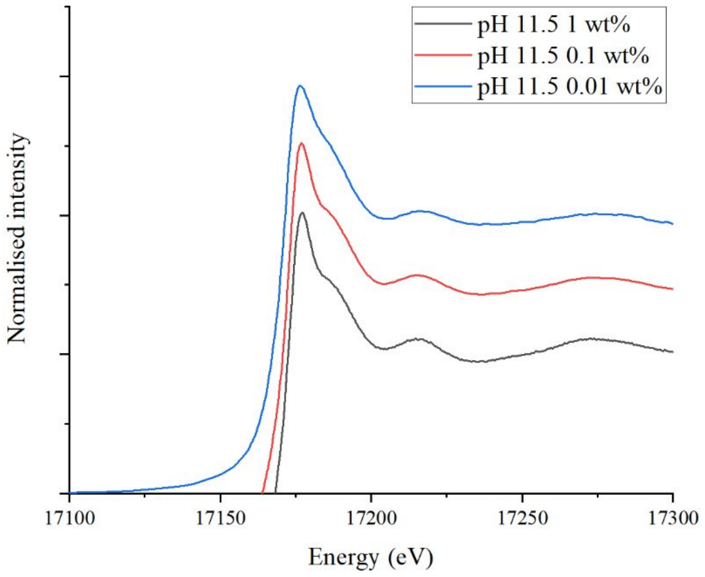

Figure S18. XANES spectra for hydrotalcite samples with U(VI) adsorbed at pH 11.5.

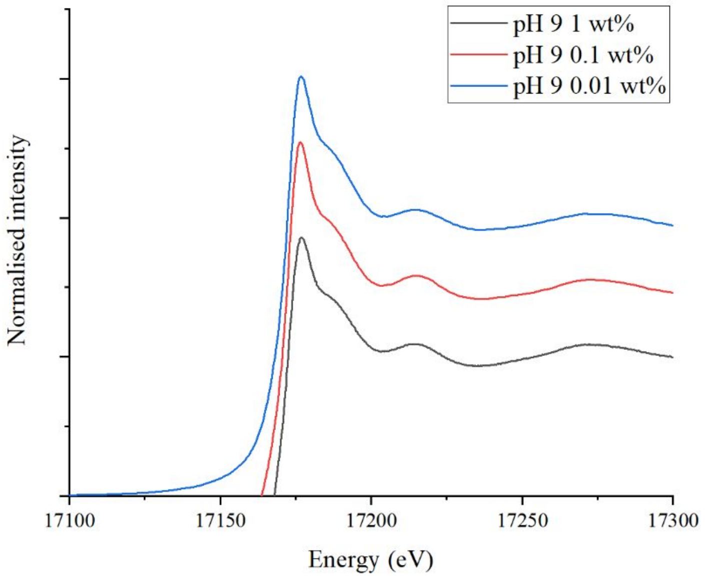

Figure S19. XANES spectra for hydrotalcite samples with U(VI) adsorbed at pH 9.

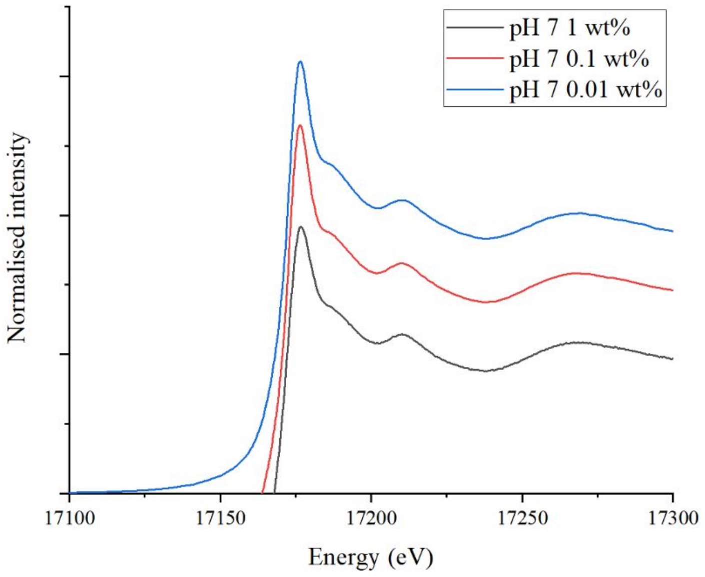

Figure S20. XANES spectra for hydrotalcite samples with U(VI) adsorbed at pH 7.

Supplementary luminescence data

Table S7. Key luminescence spectral features and the values attained from fitting of the luminescence lifetime decay profiles for samples removed from colloidal hydrotalcite of different pHs (11.5, 9 and 7) and U(VI) loading (1 wt% and 0.01 wt%).

		Spectral features (nm)	Fluorescence lifetimes (μs)
pH 11.5	1 wt%	532, 552, 574 (shoulder), 606 shoulder	69 ± 1 (24%)<br>198 ± 1 (76%)
	0.01 wt%	526, 542, 570 (shoulder)	141 ± 2 (42%)<br>303 ± 1 (58%)
pH 9	1 wt%	514, 536, 556 (shoulder), 584 (shoulder)	137 ± 2 (47%)<br>253 ± 2 (53%)
	0.01 wt%	482, 502, 522, 544	137 ± 2 (21%)<br>676 ± 22 (79%)
pH 7	1 wt%	482, 502, 522, 548, 570, 598	688 ± 15 (24%)<br>1257 ± 10 (76%)
	0.01 wt%	486, 506, 526, 554, 576, 604	493 ± 8 (18%)<br>1189 ± 7 (82%)

Supplementary PHREEQC modelling

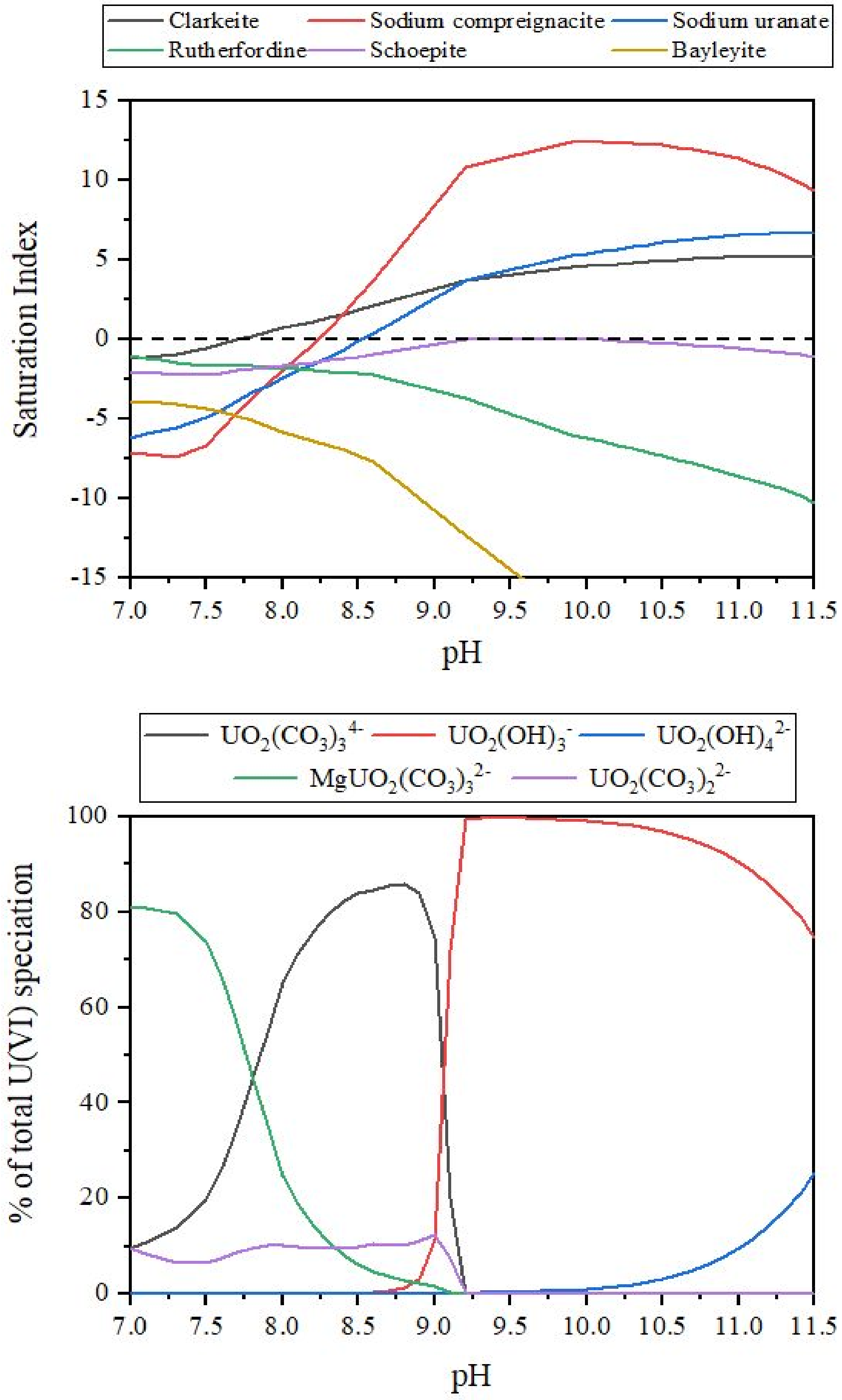

Figure S21. Top. Saturation indices of several U(VI) phases with pH for a 0.21 mM U system in equilibrium with hydrotalcite and atmospheric CO_2. **Bottom.** Speciation of U(VI) with pH for a 0.21 mM U system (1 wt% loading) in equilibrium with hydrotalcite.

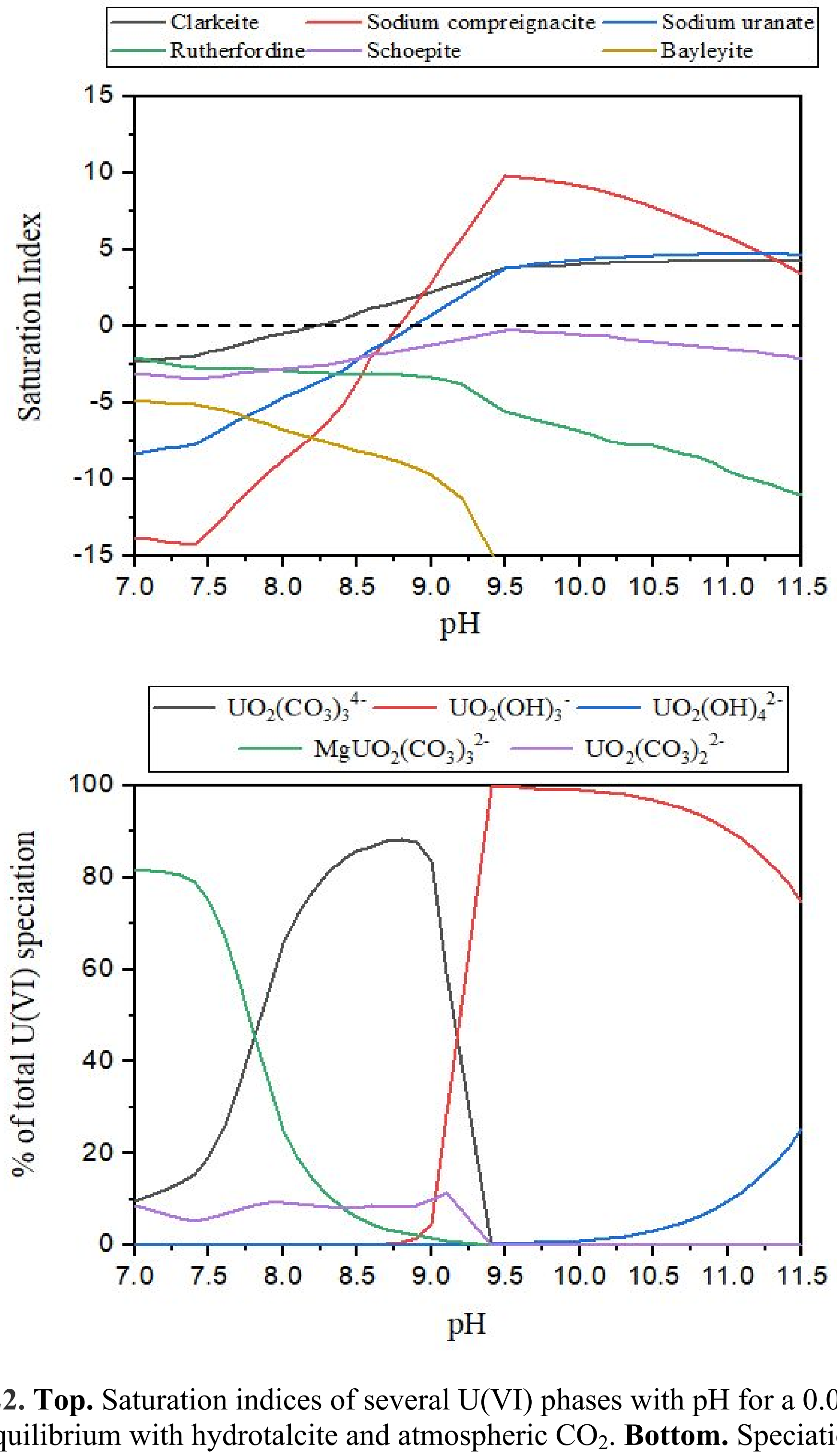

Figure S22. Top. Saturation indices of several U(VI) phases with pH for a 0.021 mM U system in equilibrium with hydrotalcite and atmospheric CO_2. **Bottom.** Speciation of U(VI) with pH for a 0.021 mM U system (0.1 wt% loading) in equilibrium with hydrotalcite.

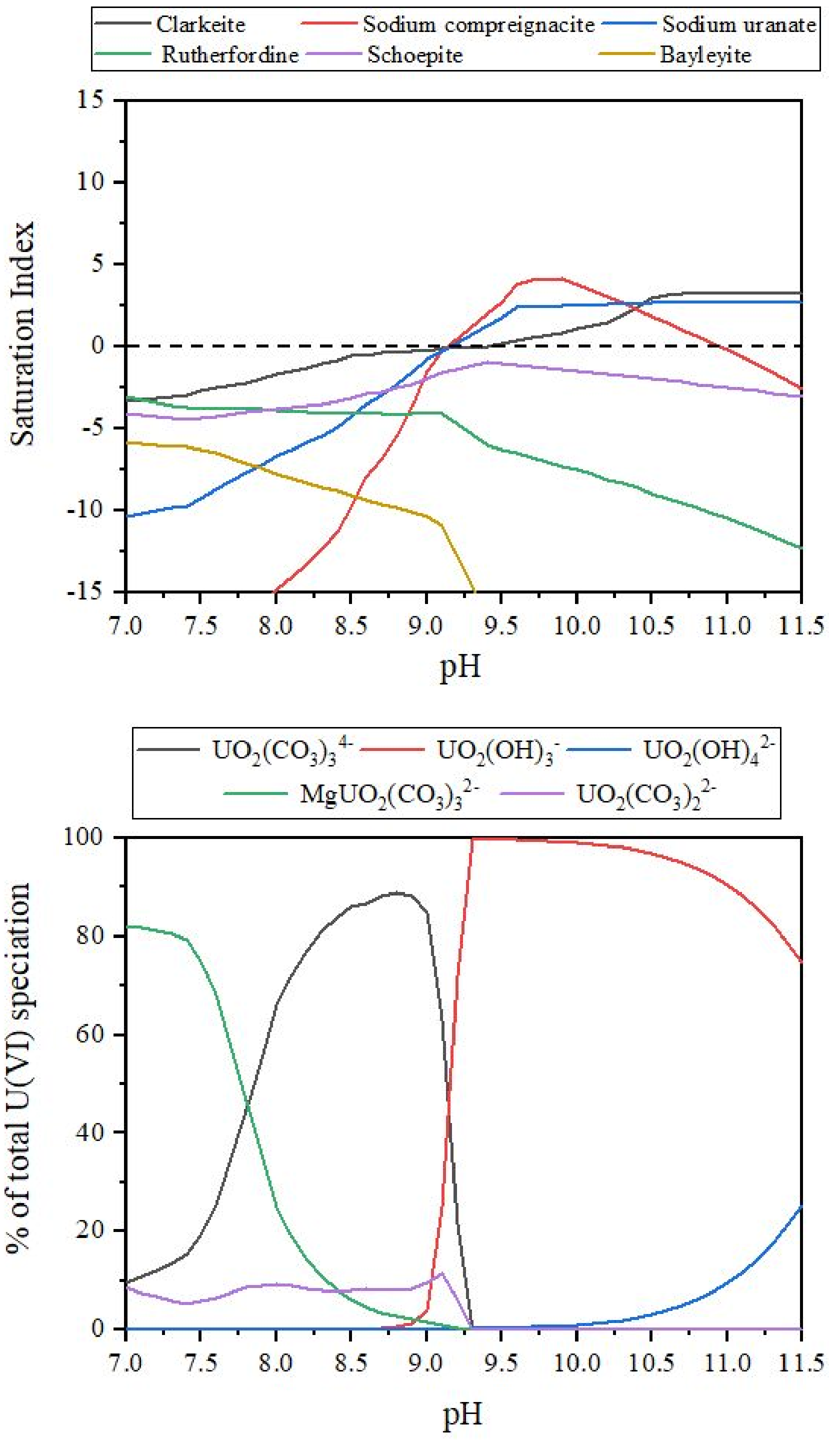

Figure S23. Top. Saturation indices of several U(VI) phases with pH for a 0.0021 mM U system in equilibrium with hydrotalcite and atmospheric CO_2. **Bottom.** Speciation of U(VI) with pH for a 0.0021 mM U system (0.01 wt% loading) in equilibrium with hydrotalcite.

Supplementary TEM images

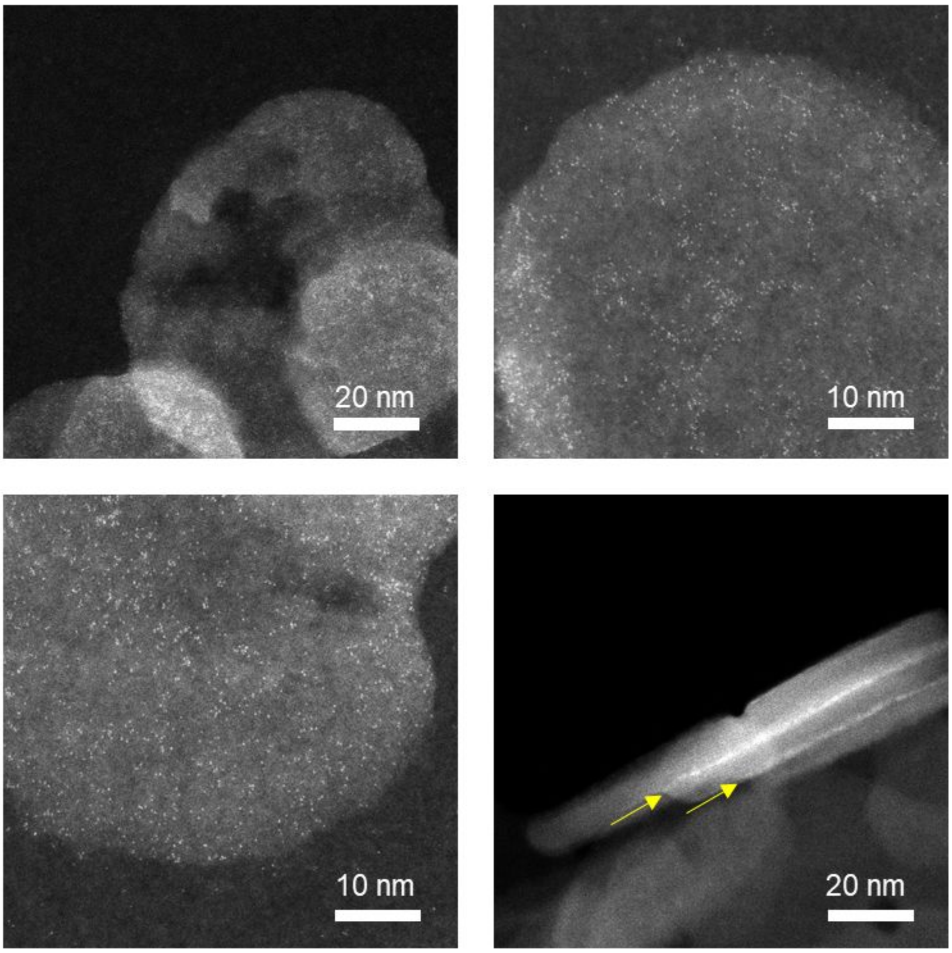

Figure S24. HAADF STEM images of hydrotalcite nanoparticles collected from a colloidal system with a U(VI) loading of 1 wt% and at pH 7.
